# Transcriptional CDK inhibitors, CYC065 and THZ1 promote Bim-dependent apoptosis in primary and recurrent GBM through cell cycle arrest and Mcl-1 downregulation

**DOI:** 10.1038/s41419-021-04050-7

**Published:** 2021-08-03

**Authors:** Viktorija Juric, Lance Hudson, Joanna Fay, Cathy E. Richards, Hanne Jahns, Maïté Verreault, Franck Bielle, Ahmed Idbaih, Martine L. M. Lamfers, Ann M. Hopkins, Markus Rehm, Brona M. Murphy

**Affiliations:** 1 Department of Physiology and Medical Physics, Royal College of Surgeons in Ireland, University of Medicine and Health Sciences, Dublin, Ireland; 2 Department of Surgery, Royal College of Surgeons in Ireland, University of Medicine and Health Sciences, RCSI Education and Research Centre, Smurfit Building, Beaumont Hospital, Dublin, Ireland; 3 Department of Pathology, Royal College of Surgeons in Ireland, University of Medicine and Health Sciences, Beaumont Hospital, Dublin, Ireland; 4 Department of Molecular Medicine, Royal College of Surgeons in Ireland, University of Medicine and Health Sciences, Beaumont Hospital, Dublin, Ireland; 5 Pathobiology Section, School of Veterinary Medicine, University College Dublin, Dublin, Ireland; 6 INSERM U 1127, CNRS UMR 7225, Sorbonne Universités, UPMC Univ Paris 06 UMR S 1127, Institut du Cerveau, Paris, France; 7 AP-HP, Hôpitaux Universitaires La Pitié Salpêtrière–Charles Foix, Service de Neurologie 2-Mazarin, Paris, France; 8 Department of Neurosurgery, Brain Tumor Center, Erasmus MC, Rotterdam, the Netherlands; 9 Institute of Cell Biology and Immunology, University of Stuttgart, Stuttgart, Germany; 10 Stuttgart Research Center Systems Biology, University of Stuttgart, Stuttgart, Germany

**Keywords:** Cancer models, Apoptosis, RNAi

## Abstract

Activation of cyclin-dependent kinases (CDKs) contributes to the uncontrolled proliferation of tumour cells. Genomic alterations that lead to the constitutive activation or overexpression of CDKs can support tumourigenesis including glioblastoma (GBM), the most common and aggressive primary brain tumour in adults. The incurability of GBM highlights the need to discover novel and more effective treatment options. Since CDKs 2, 7 and 9 were found to be overexpressed in GBM, we tested the therapeutic efficacy of two CDK inhibitors (CKIs) (CYC065 and THZ1) in a heterogeneous panel of GBM patient-derived cell lines (PDCLs) cultured as gliomaspheres, as preclinically relevant models. CYC065 and THZ1 treatments suppressed invasion and induced viability loss in the majority of gliomaspheres, irrespective of the mutational background of the GBM cases, but spared primary cortical neurons. Viability loss arose from G2/M cell cycle arrest following treatment and subsequent induction of apoptotic cell death. Treatment efficacies and treatment durations required to induce cell death were associated with proliferation velocities, and apoptosis induction correlated with complete abolishment of Mcl-1 expression, a cell cycle-regulated antiapoptotic Bcl-2 family member. GBM models generally appeared highly dependent on Mcl-1 expression for cell survival, as demonstrated by pharmacological Mcl-1 inhibition or depletion of Mcl-1 expression. Further analyses identified CKI-induced Mcl-1 loss as a prerequisite to establish conditions at which the BH3-only protein Bim can efficiently induce apoptosis, with cellular Bim amounts strongly correlating with treatment efficacy. CKIs reduced proliferation and promoted apoptosis also in chick embryo xenograft models of primary and recurrent GBM. Collectively, these studies highlight the potential of these novel CKIs to suppress growth and induce cell death of patient-derived GBM cultures in vitro and in vivo, warranting further clinical investigation.

## Introduction

Glioblastoma (GBM) is the most common primary brain tumour in adults. Despite efforts to combat this disease with an aggressive standard of care (SOC) protocol, including surgery, radiotherapy and temozolomide (TMZ) chemotherapy [1, 2] patient survival remains low, with less than 5% of those diagnosed surviving longer than 5 years [3]. In addition, almost all patients suffer from disease recurrence within 6–8 months of treatment onset and no SOC is established for those patients.

Cyclin-dependent kinases (CDKs) and their associated regulatory subunits—cyclins—are the main controllers of cell-cycle progression. Besides their well-known and studied role in cell cycle regulation, CDKs also play key physiological roles in transcription regulation [4]. Overexpression of CDKs is a well-known hallmark of many tumours [5], including GBM and can lead to uncontrolled cellular proliferation and tumour progression [6]. Therefore, inhibiting this family of enzymes has emerged as a promising strategy in the treatment of both haematologic and solid malignancies [5, 7]. Several drugs exclusively targeting CDK4/6 have been clinically approved for the treatment of specific breast cancer subtypes [8–10]. However, while preclinical studies using CDK4/6 inhibitors in cell lines and animal models of GBM have yielded positive results [11–13], clinical trials of these CDK inhibitors (CKIs) in glioma patients have not proven as successful, reviewed in [13].

First-generation CKIs such as Roscovitine (CYC202) have a broad target range [14]. Its use in an orthotopic patient-derived xenograft (PDX) model of GBM showed moderate efficacy, however, the dosing regimen required to maintain peak levels of Roscovitine in the brain resulted in toxic side-effects which would preclude its clinical utilisation [15]. Such nonspecific activity of first-generation CKIs and resulting toxicity in patients has led to the development of second-generation CKIs [16]. These have reduced off-target activities as they selectively inhibit a smaller subset of CDKs.

CYC065 is an intravenously and orally available inhibitor [17] that has reached early phase clinical trials in refractory/relapsed acute myeloid leukaemia (AML), myelodysplastic syndromes (MDS) (NCT04017546) and chronic lymphocytic leukaemia (CLL) (NCT03739554) patients. CYC065 primarily targets CDK9/2 and in comparison to its first-generation parent compound, Roscovitine, has significantly improved metabolic stability, efficacy and potency in vitro and in vivo [15, 17]. We have previously shown that CYC065 crosses the blood-brain barrier and reduces tumour growth in an orthotopic mouse model of GBM [15]. A recent trial further highlights that CYC065 is well tolerated in patients with advanced lymphomas or solid tumours (NCT02552953).

Inhibition of tumour cell transcriptional activity has been also attempted using a covalent inhibitor of CDK7, THZ1. Anti-tumour activity is evident in preclinical models of acute T-cell leukaemia [18], multiple myeloma [19], MYCN-amplified neuroblastoma [20], small cell lung cancer [21], triple-negative breast cancer [22] and colorectal cancer [23]. THZ1 has also shown promise in recent preclinical studies as a potential treatment for high-grade glioma [24] and GBM [25, 26]. THZ1, therefore, adds another potential opportunity to pharmacologically combat GBM but requires further validation with respect to response heterogeneities and treatment efficacies in well-controlled state of the art GBM model systems.

We herein comprehensively analysed the effectiveness and mechanism of action of CYC065 and THZ1 in relevant preclinical models of GBM—a panel of ten patient-derived gliomasphere cultures from both primary and recurrent tumours, carrying a range of driver mutations. Our results demonstrate that CYC065 and THZ1 inhibit tumour cell growth and induce caspase-dependent apoptotic cell death, associated with the downregulation of the anti-apoptotic protein Mcl-1. Indeed, the examined GBM models appeared to heavily depend on Mcl-1 expression for survival, as tested by protein depletion and pharmacological inhibition. Treatment efficacies were further confirmed in a semi in vivo chick embryo xenograft models.

Collectively, these data demonstrate that CYC065 and THZ1 display high anti-cancer activity in primary and recurrent GBM and provide a scientific rationale for the further development of CDK inhibitors for potential clinical utilisation in the future.

## Materials and methods

### Cell lines and cell culture

Human glioma cell lines U343 and U87 derived from primary grade III/IV gliomas were obtained from the American Type Cell Culture (ATCC, Rockville, MD, US). U343 and U87 cell lines were grown as a monolayer in DMEM (Lonza, Lisburn, UK) with heat-inactivated foetal bovine serum (10%), penicillin/streptomycin (100 U/mL) (Sigma-Aldrich, Arklow, Ireland) and maintained in a humidified incubator at 37 °C and 5% CO_2_.

Six primary patient-derived GBM cultures (N16-0125, N15-0661, N15-1027, N14-1208, N15-0385, and N16-0240) were established by Gliotex group at the Paris Brain Institute (ICM), Paris. Two primary (GTCC-6 and GTCC-7) and two recurrent (GTCC-9, GTCC-10) patient-derived cultures were generated at Erasmus Medical Centre (EMC), Rotterdam. GBM tissue samples were provided by the neuropathology laboratory of Pitie-Salpetriere University Hospital (Paris, France) and the Department of Neurosurgery of the ErasmusMC (Rotterdam, The Netherlands), and obtained as part of routine resections from patients under their informed consent (ethical approval numbers AC-2013-1962, MEC-2013-090). Patient-derived GBM cells were cultured in DMEM-F12 medium containing B27 supplement 50X (2%) (Gibco Life Technologies, Dún Laoghaire, Ireland), human bFGF (20 ng/mL), human EGF (20 ng/mL) (PeproTech EC Ltd, London, UK), penicillin/streptomycin (100 U/mL) (Sigma-Aldrich, Arklow, Ireland), heparin (5 µg/mL, Alfa Aesar, Heysham, UK) and maintained in a humidified incubator at 37 °C and 5% CO_2_. Cells were grown in extracellular matrix (ECM, 1:100; Cultrex, PathClear, Trevigen, MD, US) coated flasks as a monolayer or non-coated flasks as gliomaspheres and maintained at 37 °C in 5% CO_2_. Cells were routinely tested for mycoplasma infection and were mycoplasma free. For the cell growth curve, cells were dissociated using Accutase (Thermo Fisher Scientific, Waltham, MA, US) and seeded into six-well cell culture plates (80,000 cells/well) gliomaspheres. Cells were counted each day using a Countess automated cell counter (Thermo Fisher Scientific, Waltham, MA, US) and growth curves were determined from live-cell numbers over a 144 h period using exponential growth curves (GraphPad Software Inc., La Jolla, CA, US).

Mouse primary cortical neurons were kindly provided by Dr Orla Watters (Department of Physiology & Medical Physics, Royal College of Surgeons in Ireland, Dublin 2, Ireland). Primary cortical neurons were prepared from C57BL/6 mice as described previously [27]. Cortical neurons were seeded at a density of 30,000 cells/well in the 96-well plates precoated with 0.1 mg/mL poly-L-lysine (Sigma-Aldrich, Arklow, Ireland). The cortical neurons were plated in MEM (Gibco Life Technologies, Dún Laoghaire, Ireland) containing 10% horse serum, 10% FBS, 100 U/mL penicillin/streptomycin, 0.25% Glutamax, 0.6% glucose, 0.22% NaHCO_3_ and 1 mM pyruvic acid (Gibco Life Technologies, Dún Laoghaire, Ireland) for 4 h before replacing with serum-free Neurobasal Plus medium (Gibco Life Technologies, Dún Laoghaire, Ireland) containing 0.25% Glutamax (100X), 10 µg/mL Gentamicin and 2% B27-plus (50X) (Gibco Life Technologies, Dún Laoghaire, Ireland). The cortical neurons were maintained in culture for at least 7 days before being used for further experiments with half medium change every three days, and 1 µM cytosine β-D-arabinofuranoside (AraC) (Sigma-Aldrich, Arklow, Ireland) added at DIV4 to minimise glial proliferation. All animal work was performed with ethical approval by the RCSI Research Ethics Committee (REC1559).

### WST-1 cell viability assay

U87 and U343 cells were plated as monolayers in 96-well plates (4000 cells/well) and treated with indicated concentrations of CYC065 (#HY-101212, Medchemexpress, NJ, US) and THZ1 (#HY-80013, Medchemexpress, NJ, US) for 72 h. Patient-derived GBM cultures were plated in 96-well plates as gliomaspheres (3000 cells/well) and treated with indicated concentrations of CYC065, THZ1 and/or TMZ (#T2577, Sigma-Aldrich, Arklow, Ireland) and S-63845 (#S8383, Selleck Chemicals, Houston, TX, US) as indicated for 72 h. Mouse primary cortical neurons were seeded in flat-bottom 96-well plates coated with poly-L-lysine as 30,000 cells/well and treated with increasing concentrations of CYC065 and THZ1 for 96 h. Following treatment, WST-1 reagent (Sigma-Aldrich, Arklow, Ireland) was added in 1:10 final dilution, according to the manufacturer’s instructions. WST-1 salt is cleaved to a soluble formazan dye by a NAD(P)H-dependent reaction in viable cells. Plates were incubated for 2 h in a humidified incubator at 37 °C and 5% CO_2_ and the absorbance of each sample was measured at 450 and 620 nm using a microplate reader (GENios, Tecan, Weymouth, UK). Background signal (620 nm) was then subtracted from the 450 nm reads. The absorbance was proportional to the number of viable cells and expressed relative to DMSO (0.1%) control-treated groups.

### Fluorometric cell viability and cytotoxicity detection

Gliomaspheres were seeded as 3000 cells/well in 96-well plates and treated as indicated 24 h post-seeding. Mouse cortical neurons were seeded as 30,000 cells/well in 96-well plates for 7 days before use (as described above). Following 72 h (gliomaspheres) and 96 h (primary cortical neurons) incubation with DMSO (0.1%), CYC065 (3 μM) and THZ1 (100 nM), gliomaspheres and mouse primary cortical neurons were stained with Calcein AM (Invitrogen, Waltham, Massachusetts, US). In total, 4 μM Calcein AM in DPBS was added to a 15 mL tube, mixed and incubated at 37 °C for 15 min. Media was then removed from the cultures and 100 µL of Calcein AM/DPBS mix was added/well for 30 min at 37 °C prior to imaging. Images were taken immediately with an Eclipse TE300 inverted microscope using the FITC channel.

### Hoechst/PI staining

U87 and U343 cells were seeded as 4000 cells/well in F-bottom 96-well plates. Cells were pre-stained with 1 µg/mL PI and 1 µg/mL Hoechst 33258 (Sigma-Aldrich, Arklow, Ireland). Images were taken with an Eclipse TE300 inverted microscope following 72 h incubation with DMSO (0.1%), CYC065 (3 μM) and THZ1 (100 nM).

### Western blot analysis

To obtain whole-cell lysates, cells were washed with Dulbecco’s phosphate-buffered saline (DPBS, Gibco Life Technologies, Dún Laoghaire, Ireland) solution and lysed on ice with a RIPA lysis buffer containing 150 mM NaCl, 0.1% Triton X-100, 0.5% sodium deoxycholate, 0.1% sodium dodecyl sulfate (SDS), 50 mM Tris in ddH_2_O, pH 8, and protease/phosphatase inhibitor cocktails (Sigma-Aldrich, Arklow, Ireland). Protein concentrations were determined using a BCA protein assay kit (Pierce, Rockford, IL, US) following the manufacturer’s instructions. An equal amount of proteins (20 μg) were diluted with Laemmeli loading buffer and separated on 7–15% SDS-polyacrylamide gels and transferred to nitrocellulose membranes using wet transfer. Membranes were blotted with primary antibodies at the following dilutions: Cdk-2 (#sc-6248, 1:500), Cdk-7 (#sc-7344, 1:500), Cdk-9 (#sc-13130, 1:500) (Santa Cruz Biotechnology, Santa Cruz, CA, US), Mcl-1 (#94296, 1:1000), cleaved caspase-3 (#9661, 1:1000), caspase-3 (#9662, 1:1000), cleaved caspase-7 (#9491, 1:1000), caspase-7 (#9494, 1:1000), cyclin B1 (#4135, 1:1000), p-CDK-1 (#9111, 1:1000), Bim (#2933, 1:1000) (Cell Signalling, Danvers, MA, US), RNA Poll II (#39097, 1:1000, Active Motif, Carlsbad, CA, US), RNA Pol II CTD p-Ser-2 (#04-1571, 1:1000), RNA Pol II CTD p-Ser-5 (#04-1572, 1:1000) (Merck KGaA, Darmstadt, Germany), ß-actin (#A5441, 1:5000), α-tubulin (#T-6195, 1:5000) (Sigma-Aldrich, Arklow, Ireland) and GAPDH (#MAB374, 1:5000, Merck KGaA, Darmstadt, Germany). Membranes were next incubated with rat (#31470, Thermo Fisher Scientific, Waltham, MA, US), mouse (#AP124P, 1:5000) or rabbit (#AP132P, 1:5000) (Merck KGaA, Darmstadt, Germany) horseradish peroxidase-conjugated secondary antibodies and protein bands were visualised using Supersignal West Pico Chemiluminescent Substrate (Pierce, Rockford, IL, US). Images were captured using Fuji-film LAS-4000 (Fuji, Sheffield, UK). Densitometry analysis was done using Image Studio Lite v5.2 (LI-COR Biosciences Ltd., UK).

### Cell death analysis using flow cytometry

Cell death was measured using a BD LSRII flow cytometer (BD Biosciences, Oxford, UK). U87 and U343 cells were plated as monolayers in a 6-well plate (500 000 cells/well). GBM PDCLs were seeded as gliomaspheres, 300 000 cells per well in a 6-well plate. After 24 h cells were treated with DMSO (0.1%), 3 μM CYC065 or 100 nM THZ1 for 72 or 120 h. For caspase dependence experiments, cells were pre-treated with 50 μM QVD-OPh (Selleckchem, Houston, TX, USA) for 1 h and then treated with 3 μM CYC065 or 100 nM THZ1 for 72 h. U87 and U343 cells were detached following 72 h treatment using Trypsin-EDTA solution (Sigma-Aldrich, Arklow, Ireland) for 4 min at 37 °C and 5% CO_2_. Following treatments, gliomaspheres were pelleted, dissociated using Accutase (Thermo Fisher Scientific, Waltham, MA, US). Cells were next incubated in 100 μL of binding buffer (10 nM HEPES, 135 nM NaCl, 5 mM CaCl_2_, pH 7.4) containing AnnexinV-FITC conjugate (1:200) (BioVision, Mountain View, CA, USA) and propidium iodide (1:500) (Sigma-Aldrich, Arklow, Ireland) for 20 min on ice in the dark. A total of 1 × 104 gated cells were acquired. Acquired data from the flow cytometry analyses were analysed using FlowJo Software 10.6.2. Version 5.0 (Becton, Dickinson and Company, Franklin Lakes, NJ, US).

### Cell cycle analysis

All tested cell lines were cultured as described above and treated with DMSO (0.1%), 3 μM CYC065 or 100 nM THZ1 for 48, 72 or 96 h as indicated. At the end of treatment, the cells were dissociated, washed twice with ice-cold DPBS and centrifuged for 5 min at 500 g. The resulting pellets were resuspended in 1 mL ice-cold DPBS, followed by 1 mL 70% ice-cold ethanol (Sigma-Aldrich, Arklow, Ireland). The fixed cells were then kept at 4 °C overnight. 100 μL Propidium iodide (PI) solution containing 50 μg/mL PI, 100 μg/mL RNaseA (Thermo Fisher Scientific, Waltham, MA, US) and 0.05% Triton-X (Sigma-Aldrich, Arklow, Ireland) was added to the cell pellets for one hour at 37 °C. A total of 1 × 104 gated cells were acquired. Data were analysed using FlowJo cell cycle analysis.

### siRNA transfection

Control siRNA or siRNA targeting the MCL-1 or BIM were transiently transfected into N16-0240 and GTCC-9 cells using Lipofectamine 2000 transfection reagent (Thermo Fisher Scientific, Waltham, MA, US) according to the manufacturer instructions. Briefly, cells were seeded as 2-D cultures in 6-well plates coated with ECM (300 000 cells/well) and transfected with 20 nM of Mcl-1 siRNA, 30 nM of Bim siRNA or 20 or 30 nM of control siRNA, respectively (Thermo Fisher Scientific, Waltham, MA, US) in Opti-MEM (Gibco Life Technologies, Dún Laoghaire, Ireland). Twenty-four hours post-transfection cells were detached and reseeded in DMEM-F12 complete medium in non-coated plates as described above and incubated for an additional 24 h. Whole-cell lysates were collected as described previously and transfection efficacy was determined using Western blot analysis. Cells with depleted Bim were then treated with DMSO (0.1%), 3 μM CYC065 or 100 nM THZ1 for 24 h and the percentage of apoptotic cells was determined using flow cytometry, as described above.

### Colony formation assay

600 cells/well were seeded in 6-well plates coated with extracellular matrix (ECM). Treatments were applied for 72 h and media was replaced with fresh media. Cells were left to recover for 12 days. Cells were washed with DPBS and fixed using methanol and acetic acid solution (Sigma-Aldrich, Arklow, Ireland) for 5 min followed by 30 min incubation in crystal violet (1:100 in ddH_2_O) (Abcam, Cambridge, UK). Wells were washed with ddH_2_O and dried, and colonies were counted using Fiji/ImageJ software (version 1.52n, U.S. National Institutes of Health, Bethesda, Maryland, MD, US).

### Gliomasphere invasion assay

Gliomaspheres were cultured as described. Following gliomasphere formation 500 μL cell suspension was allowed to settle in Eppendorf tubes on ice. The supernatant was removed and spheres mixed with extracellular matrix protein (0.4 mg/mL final concentration, ECM) diluted in DMEM-F12 medium (without growth factors), then seeded in triplicate in 96 well-plates and treated as indicated (0.1% DMSO, 3 μM CYC065 or 100 nM THZ1). Plates were imaged immediately every hour for 24 h using a Cell Discoverer 7 microscope (Carl Zeiss, Germany). Data were analysed using Fiji/ImageJ software.

### Semi-in vivo chick embryo xenograft model

Semi-in vivo chorioallantoic membrane (CAM) assays were carried out as described previously [28, 29]. Fertilised white chicken eggs were obtained from Shannon Vale Foods, Cork, Ireland. Eggs were incubated in a humidified Napco Incubator at 37 °C. A small window was made in the egg shell on day 3 of chick embryo development under aseptic conditions. The window was resealed with semi-permeable adhesive tape and eggs were returned to the incubator until day 8 of chick embryo development. On day 8, 2 × 10^6^ GBM tumour cells (N16-0240 or GTCC-9) were resuspended in 50 μL DMEM-F12 medium (no supplements added) and 50 μL Matrigel (Corning, Bedford, MA, US) and implanted on top of the CAM within a silicon ring. Eggs were resealed and placed in the incubator for 48 h. On day 10, tumours were topically treated with DMSO control (0.01%), 3 μM CYC065, 100 nM THZ1 diluted in DMEM-F12 (no supplements added), then resealed and returned to the incubator. On day 14 tumours were excised with their surrounding CAM, fixed in 4% (w/v) paraformaldehyde (PFA), paraffin embedded and cut in 5 μm sections. Ethical approval was obtained by the RCSI Research Ethics Committee (REC202003011).

### Immunohistochemistry

Chick embryo tumour xenograft sections were deparaffinised and immunohistochemically stained for cleaved caspase-3 (#9661, Cell Signalling, Danvers, MA, US) and Ki67 (clone MIB-1) (#M724029-2, Agilent, Santa Clara, CA, US) (performed on an auto-stainer in the histopathology department, RCSI). Images were taken using an Olympus BX51 microscope (20x).

### TCGA data analysis

Tumour gene expression and clinical data including patient subtype information were obtained from the publicly available database, The Cancer Genome Atlas (TCGA) [30]. TCGA GBM dataset was accessed through the open-access GlioVis portal [31].

### Statistical analysis and software

All data from Western blotting were representative of at least three independent experiments with samples collected from at least three biological replicates. Statistical analyses were performed using GraphPad Prism software version 8.4.3 (GraphPad Software Inc., La Jolla, CA, US). The IC50 value was calculated using nonlinear regression analysis in Prism 8.4.3. Results are presented as mean ± SEM. Data were tested for significance using appropriate tests as detailed in the respective figure legends (*denotes *p* values < 0.05, **denotes *p* values < 0.01, ***denotes *p* values < 0.001, ****denotes *p* values < 0.0001 and were considered to be statistically significant; ns = not significant). Webb’s fractional product method [32] was used to calculate the synergy between CYCO65/THZ1 and TMZ where scores <0.9 were considered as synergistic. BioRender (BioRender.com) was used to generate Figs. 5A and 6.

## Results

### CYC065 and THZ1 treatments cause viability loss in patient-derived gliomaspheres while sparing neuronal cells

**Fig. 1:**
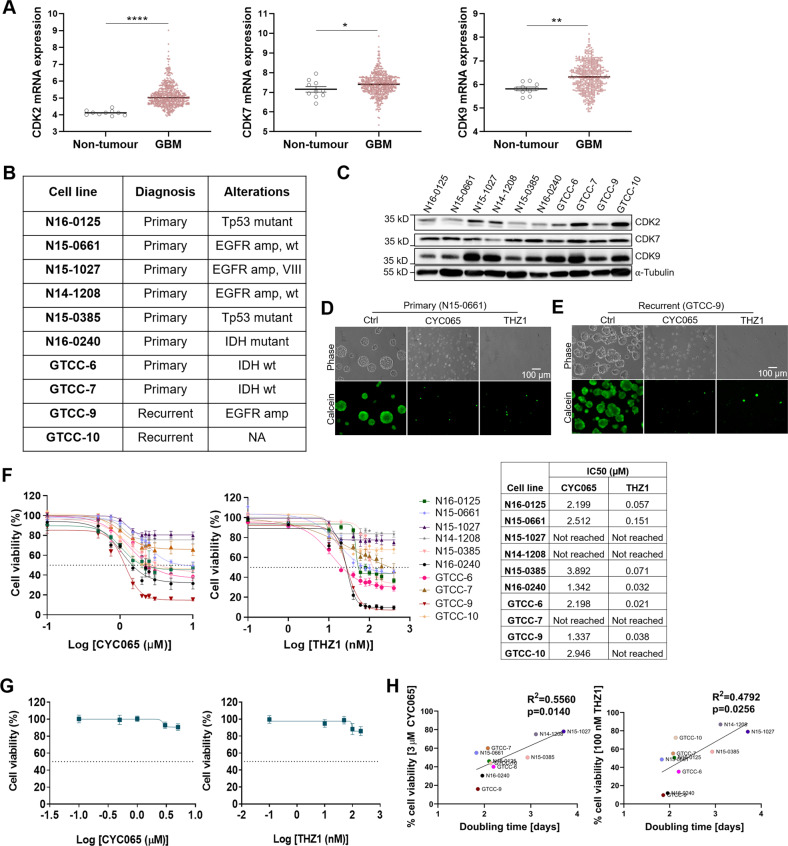
Targeting CDK2/9 or 7 in GBM causes loss of cell viability whilst sparing neuronal cells. **A** Publicly available TCGA data were analysed to study mRNA expression of *CDK2, 7* and *9* in GBM patient tumour tissues compared to non-tumour tissue. A two-tailed unpaired *t*-test was used to determine significance, whereby ****p < 0.0001; **p < 0.01; *p < 0.05. Data from a total of 538 samples were analysed. **B** Molecular characteristics of ten patient-derived GBM patient-derived cultures used in this study. **C** CDK2, 7, and 9 protein expression levels in 3-D culturing system were analysed using Western blot. α-Tubulin was used as a loading control. Western blot analysis is performed in *n* = 3 biological replicates and representative blots are shown here. **D** Gliomasphere morphology and viability was followed using Calcein-AM staining 72 h post-treatment with DMSO control, 3 μM CYC065 and 100 nM THZ1 in primary gliomasphere culture—N15-0661. **E** Gliomasphere morphology and viability was followed using Calcein-AM staining 72 h post-treatment with DMSO control, 3 μM CYC065 and 100 nM THZ1 in recurrent gliomasphere culture—GTCC-9. **D**, **E** Images were taken with an Eclipse TE300 inverted microscope (scale bar = 100 μm). *N* = 3 independent experiments performed in triplicate for each condition. **F** Cell viability was measured 72 h post-treatment with increasing concentrations of CYC065 and THZ1 in patient-derived gliomaspheres using WST-1 viability assay. Corresponding IC50 values are given in the table. Data are expressed as mean ± SEM. *N* = 3 independent experiments performed in triplicate. **G** Cell viability was determined using WST-1 viability assays in mouse primary cortical neurons 96 h post-treatment with increasing concentrations of CYC065 and THZ1. Data are expressed as mean ± SEM. *N* = 3 independent experiments performed in triplicate. **H** Correlation between gliomasphere cell doubling time and treatment response to 3 μM CYC065 and 100 nM THZ1 was calculated using Pearson correlation coefficient. *N* = 3 independent experiments performed.

CDK overexpression is evident in many tumours, including GBM [33]. The analysis of the TCGA GBM dataset revealed increased mRNA expression of *CDK2, 7*, and *9* in GBM patient tumours compared to non-tumour tissue (Fig. 1A). The expression did not differ between different GBM subtypes (Supplemental Fig. 1), highlighting that these CDKs are potential therapeutic targets across all GBM subtypes. To assess the anti-tumour potential of CYC065 and THZ1 in GBM, we initially tested two well-characterised cell lines, U87 and U343. Both CYC065 and THZ1 substantially reduced cell viability and increased apoptotic cell death in these cell lines (Supplemental Fig. 2A–C). To extend our findings, we next established a panel of ten patient-derived cultures from both primary and recurrent GBM patients and also capturing frequent driver mutations (Fig. 1B, Supplemental Fig. 3). While CDK7 was expressed largely uniformly across all gliomasphere cultures, CDK2 and 9 expression appeared to be highly heterogeneous (Fig. 1C).

As one of the main reasons for GBM recurrence is incomplete surgical resection due to invasion of surrounding healthy brain tissue by the tumour [34], cell invasion was followed upon treatment with CKIs in gliomaspheres embedded in extracellular matrix. CKIs suppressed cell invasion, noticeably already 24 h following treatment onset (Supplemental Fig. 4A–D). CYC065 and THZ1 single-agent treatments reduced size and viability in both primary, N15-0661 (Fig. 1D) and recurrent, GTCC-9 (Fig. 1E) gliomasphere cultures, indicating that CKIs are potent and penetrate the spheres. A more detailed analysis of cell viability upon exposure to increasing concentrations of CYC065 and THZ1 highlighted that most gliomasphere cultures responded strongly to CYC065 and THZ1 treatment with IC50 values in the low µM or nM range, respectively (Fig. 1F). The observed differences in responses were not related to known EGFR, IDH1 and Tp53 mutations nor did they correlate with CDK2, 7 and 9 protein expression (Fig. 1B, Supplemental Fig. 5). In contrast, CKIs did not affect the viability of untransformed mouse primary cortical neurons or neurite outgrowth during four days of treatment (Fig. 1G, Supplemental Fig. 6).

Due to attempts in combining CDK inhibitors with SOC to improve patient outcomes [13], we investigated whether combining CKIs with TMZ would result in synergistic effects due to the CYC065’s direct and THZ1’s indirect regulation of CDK2, which otherwise might counteract TMZ-induced DNA damage by promoting the DNA damage repair [35]. The vast majority of gliomasphere cultures were highly resistant to TMZ regardless of their MGMT status (Supplemental Fig. 7A). However, combining CYC065 or THZ1 with TMZ failed to synergize with or potentiate treatment responses in gliomasphere cultures (Supplemental Fig. 7B, C). Therefore, our further studies focused on using CYC065 and THZ1 as single agents. Due to their involvement in cell cycle regulation, we investigated whether CKI-treatment response correlates with proliferation velocities. Indeed, responsiveness strongly correlated with cell doubling times (Fig. 1H). Altogether, these data demonstrate that CYC065 and THZ1 preferentially target fast proliferating GBM cells whilst sparing primary neuronal cells.

### CYC065 and THZ1 induce caspase-dependent apoptosis and suppress long-term proliferative capacity in responsive gliomaspheres

**Fig. 2:**
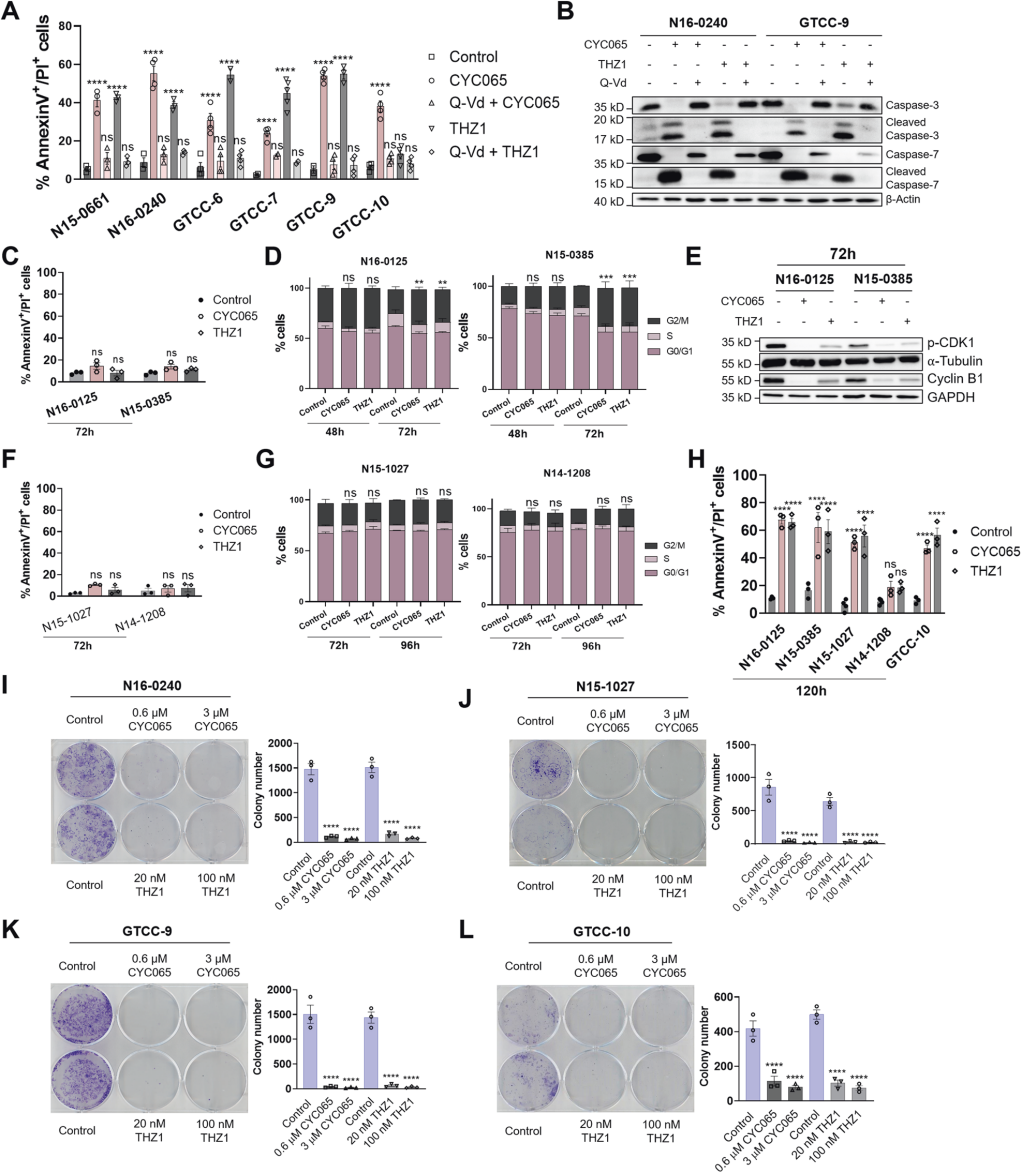
CYC065 and THZ1 induce apoptosis and suppress long-term proliferative capacity in responsive gliomaspheres. **A** Flow cytometry was used to assess the number of AnnexinV^+^/PI^+^ cells in GBM cultures treated with DMSO, 3 μM CYC065 or 100 nM THZ1 for 72 h in the presence or absence of the pan-caspase inhibitor QVD-OPh (in figure Q-Vd) (50 µM, 1 h pretreatment). **B** Caspase activation was followed using Western blot analysis in the primary and recurrent gliomaspheres treated as indicated for 72 h. Western blot analysis was performed for *n* = 3 biological replicates and representative blots are shown here. β-actin was used as a loading control. **C** Flow cytometry using AnnexinV/PI staining was employed to assess the number of AnnexinV^+^/PI^+^ cells following 72 h treatment with DMSO, 3 μM CYC065 or 100 nM THZ1 in N16-0215 and N15-0385 gliomasphere cultures. **D** Flow cytometry was used to assess the percentage of cells in the G0, S and G2/M phases of the cell cycle. Data from gliomaspheres permeabilized and stained with propidium iodide after 48 and 72 h treatment with DMSO, 3 μM CYC065 or 100 nM THZ1 in N16-0125 and N15-0385 cultures are shown here. **E** Western blot analysis of whole-cell lysates collected 72 h post-treatment with DMSO, 3 μM CYC065 or 100 nM THZ1 in N16-0125 and N15-0385 gliomaspheres. Expression of p-CDK1 and cyclin B1 was determined. α-Tubulin and GAPDH were used as loading controls. Western blot analysis was performed in *n* = 3 biological replicates and representative blots are shown here. **F** Flow cytometry using AnnexinV/PI staining was employed to assess the number of AnnexinV^+^/PI^+^ cells following 72 h treatment with DMSO, 3 μM CYC065 or 100 nM THZ1 in N15-1027 and N14-1208 gliomaspheres. **G** Flow cytometry was used to assess the percentage of cells in the G0, S and G2/M phases of the cell cycle. Data was collected from gliomaspheres permeabilized and stained with propidium iodide after 72 and 96 h treatment with DMSO, 3 μM CYC065 or 100 nM THZ1 in N15-1027 and N14-1208 cultures. **H** Gliomasphere cultures were treated with DMSO, 3 μM CYC065 or 100 nM THZ1 for 120 h and analysed using AnnexinV/PI staining on flow cytometry to determine the percentage of AnnexinV^+^/PI^+^ cells. **I**–**L** Colony formation assay was performed 72 h post-treatment with increasing concentrations of single CKI-treatments as indicated. Colonies were stained using crystal violet and colony number were counted in ImageJ. Data are expressed as mean ± SEM. One-way ANOVA with post-hoc Tukey’s analysis was used for statistical analysis, whereby, ****p < 0.0001; *n* = 3 independent experiments were performed in triplicate for each condition. **A**, **C**, **D**, **F**, **G**, **H** Data are expressed as mean ± SEM. Two-way ANOVA with post-hoc Tukey’s analysis was used for statistical analysis, whereby, **p < 0.01, ***p < 0.001, ****p < 0.0001, ns = not significant; *n* ≥ 3 independent experiments were performed.

We next investigated whether CKI-induced reductions in cell viability (Fig. 1F) were accompanied by induction of cell death. Treatment with either CYC065 or THZ1 for 72 h induced significant levels of apoptosis in faster proliferating primary and recurrent gliomasphere cultures, as indicated by pan-caspase inhibitor Q-Vd-OPh preventing cell death (Fig. 2A). Correspondingly, Western blot analysis showed that both CYC065 and THZ1 induced activation of executioner caspases-3 and -7 (Fig. 2B). However, among the cell lines identified as responsive (Fig. 1F), two cell lines, N16-0125 and N15-0385, failed to undergo apoptotic cell death during this treatment period (72 h, Fig. 2C). Due to the possible effects of CKIs on cell cycle progression, we hypothesised that impaired proliferation might account for the loss in viability when compared to untreated controls. Indeed, cell cycle analysis showed substantial G2/M arrest in both cell lines at this time point (Fig. 2D, Supplemental Fig. 8). This correlated with p-CDK-1 dephosphorylation and cyclin B1 loss (Fig. 2E). Similar effects on cell cycle distribution were observed in the two commercially available GBM cell lines studied here (Supplemental Fig. 2D).

In comparison, the two gliomasphere cultures, N15-1027 and 14-1208, which were classified as highly resistant in cell viability measurement (Fig. 1F) failed to undergo apoptotic cell death (Fig. 2F) and neither were arrested in their cell cycle (72 and 96 h post-treatment, Fig. 2G). Moreover, recurrent GTCC-10 gliomaspheres were responsive to CYC065 whilst not responding to THZ1 72 h post-treatment (Fig. 1F) and this was also reflected in the apoptotic cell death assay (Fig. 2A). Since we observed a strong correlation between proliferation rates and responsiveness (Fig. 1H), we hypothesised that due to slow growth treatment effects simply might require longer times to manifest. Indeed, when these gliomasphere cultures were treated with CYC065 and THZ1 for prolonged times (120 h), significant amounts of apoptotic cell death were achieved in four out of five cultures that otherwise did not show any overt signs of cell death at earlier time points (Fig. 2H). This likewise applied to GTCC-10 cells (Fig. 2H, compare to Fig. 2A).

We noted that CKI treatments can eliminate large portions of the overall cell populations, but fractions of cells apparently survived these treatments. In vivo, such fractions could drive tumour re-growth or recurrence following initial treatment. We therefore next studied if CKI treatments allow long-term survival and clonogenic outgrowth of non-responsive fractions of GBM cells. Patient-derived GBM cultures were treated with CKIs for 72 h and colony formation assays were performed. In four cultures tested, CKIs suppressed the long-term proliferation capacity in primary (Fig. 2I, J) and recurrent (Fig. 2K, L) cultures even at concentrations lower than the IC50.

Overall, this suggests that CYC065 and THZ1 treatment induce apoptotic cell death, subsequent to cell cycle arrest, in the majority of primary and recurrent gliomaspheres. Importantly, apoptosis responses required prolonged times to manifest, indicating that caution needs to be taken when classifying GBM cell isolates as responsive or resistant, especially since such patient-derived-based models have considerably slower proliferative capacity than conventional established cell lines. CKIs furthermore suppress the long term proliferation capacity of cell fractions not responding with apoptotic cell death, further supporting the broad anti-neoplastic activity of CKIs in the GBM setting.

### CYC065 and THZ1 downregulate the anti-apoptotic protein Mcl-1, which suffices to sensitize gliomasphere cultures to apoptosis

**Fig. 3:**
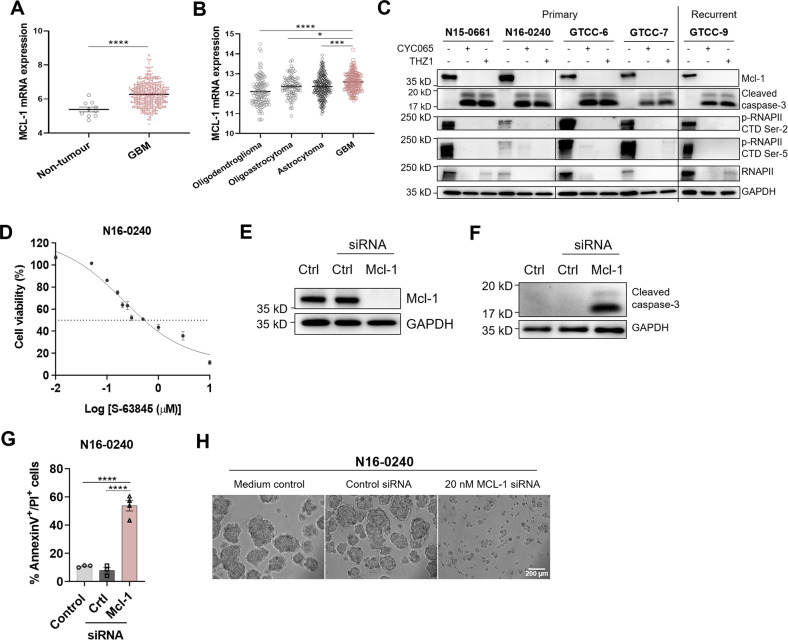
CYC065 and THZ1 downregulate Mcl-1 in the gliomaspheres which suffices the induction of apoptotic cell death in these cultures. **A** The TCGA dataset was analysed to study mRNA expression of *Mcl-1* in non-tumour tissue and GBM patient tumour tissues. A two-tailed unpaired *t*-test was used to determine significance, whereby ****p < 0.0001. Data from a total of 538 samples were analysed. **B** The TCGA data were analysed to study mRNA expression of *Mcl-1* in low-grade gliomas and GBM patient tumour tissues. One-way ANOVA with post-hoc Tukey’s analysis was used to determine significance, whereby ****p < 0.0001, ***p < 0.001, *p < 0.05. Data from a total of 667 samples were analysed. **C** Levels of Mcl-1, cleaved caspase-3, RNAPII CTD Ser2/5 phosphorylation and RNAPII in the gliomaspheres upon treatment with DMSO, 3 μM CYC065 or 100 nM THZ1 for 72 h were followed using Western blot analysis. GAPDH is used as a loading control. Western blot analysis was performed in *n* = 3 biological replicates and representative blots are shown here. **D** N16-0240 gliomasphere culture response to Mcl-1 inhibitor, S-63842 was analysed using WST-1 viability assay 72 h post-treatment. Data are expressed as mean ± SEM; *n* = 3 independent experiments performed in triplicate. **E** N16-0240 cells were transfected with scrambled control siRNA (20 nM) and Mcl-1-targeting siRNA (20 nM) for 48 h. Transfection efficiency was assessed by Western blotting and GAPDH was used as a loading control. Western blot analysis was performed in *n* = 3 biological replicates and representative blots are shown here. **F** N16-0240 cells were transfected with scrambled control siRNA (20 nM) and Mcl-1-targeting siRNA (20 nM) for 48 h. Caspase-3 activation was followed using Western blot analysis. GAPDH was used as a loading control. Western blot analysis was performed in *n* = 3 biological replicates and representative blots are shown here. **G** Apoptotic cell death was measured using AnnexinV/PI staining in cells transfected with scrambled control siRNA (20 nM) and Mcl-1-targeting siRNA (20 nM) and in N16-0240 gliomaspheres. Data are expressed as mean ± SEM. One-way ANOVA with post-hoc Tukey’s analysis was used for statistical analysis, whereby, ****p < 0.0001; *n* = 3 independent experiments. **H** Morphology changes in N16-0240 cells transfected with scrambled control siRNA (20 nM) and Mcl-1-targeting siRNA (20 nM). Images were taken with an Eclipse TE300 inverted microscope (scale bar = 200 μm). *N* = 3 independent experiments performed in triplicate for each condition.

**Fig. 4:**
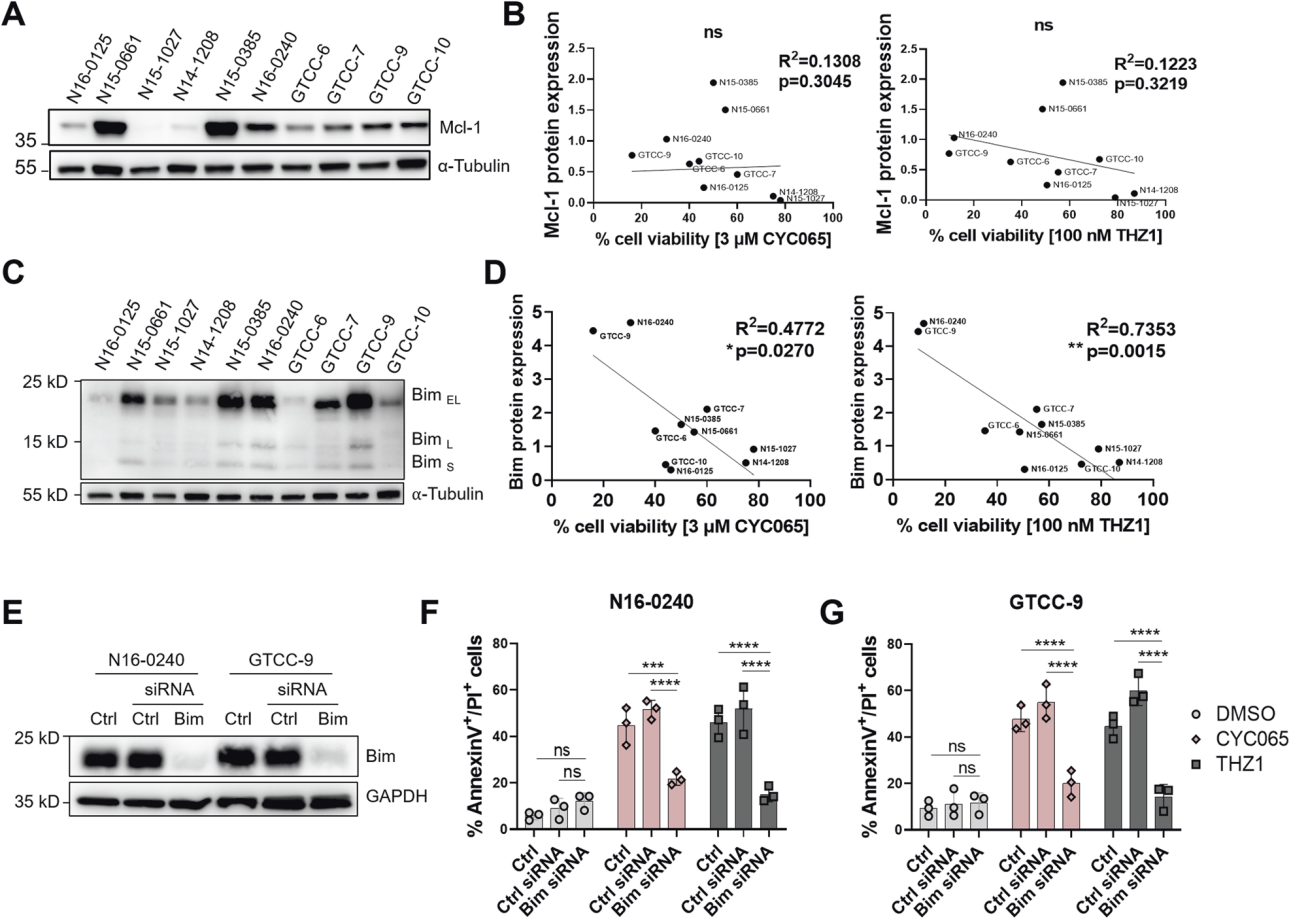
Downregulation of Mcl-1 triggers Bim-dependent apoptosis in sensitive gliomasphere cultures. **A** Whole-cell lysates from ten patient-derived gliomasphere cultures were used to determine Mcl-1 protein expression. α-Tubulin was used as a loading control. Western blot analysis is performed in *n* = 3 biological replicates and representative blots are shown here. **B** Correlation analysis of Mcl-1 basal protein expression in gliomaspheres and treatment response to 3 μM CYC065 (left panel) and 100 nM THZ1 (right panel) was done using Pearson correlation coefficient. *N* = 3 independent experiments were performed. **C** Whole-cell lysates from ten patient-derived gliomasphere cultures were used to determine Bim protein expression. α-Tubulin was used as a loading control. Western blot analysis is performed in *n* = 3 biological replicates and representative blots are shown here. **D** Correlation analysis between Bim basal protein expression in gliomaspheres and treatment response to 3 μM CYC065 (left panel) and 100 nM THZ1 (right panel) were calculated using Pearson correlation coefficient. *N* = 3 independent experiments were performed. **E** N16-0240 and GTCC-9 cultures were transfected with scrambled control siRNA (30 nM) and Bim-targeting siRNA (30 nM) for 48 h. Transfection efficiency was assessed by Western blotting and GAPDH was used as a loading control. Western blot analysis was performed in *n* = 3 biological replicates and representative blots are shown here. **F**, **G** Flow cytometry was used to assess the number of AnnexinV^+^/PI^+^ cell in cultures transfected scrambled control siRNA (30 nM) and Bim-targeting siRNA (30 nM) for 48 h and following 24 h treatment with DMSO, 3 μM CYC065 or 100 nM THZ1 in N16-0240 (**D**) and GTCC-9 (**E**) gliomaspheres. Data are expressed as mean ± SEM. Two-way ANOVA with post-hoc Tukey’s analysis was used for statistical analysis, whereby, ***p < 0.001, ****p < 0.0001, ns = not significant; *n* = 3 independent experiments performed for all experiments.

DNA damage due to TMZ treatment and also CKIs induce cell cycle arrest, which only in the case of CKIs translates into apoptosis induction in our GBM models. Since all gliomaspheres were highly resistant to the clinically achievable concentrations of TMZ but most instead responded to low concentrations of CDK inhibition, we next studied which sensitization mechanism might contribute to the CKI responsiveness. The Bcl-2 protein family regulates apoptotic engagement of mitochondria, with the subgroup of anti-apoptotic family members (Bcl-2, Bcl-xL, Mcl-1 as the main players) able to confer apoptosis resistance [36, 37]. We therefore initially examined the expression of *Bcl-2-*, *Bcl-xL* and *Mcl-1*, of which Mcl*−1* but not *Bcl-2* or *Bcl-xL* were significantly upregulated in GBM compared with non-tumour tissue (Fig. 3A, Supplemental Fig. 9). Moreover, *Mcl-1* mRNA amounts increased from lower-grade gliomas to grade IV GBM (Fig. 3B). Among the anti-apoptotic members of the Bcl-2 family, Mcl-1 is particularly short-lived [38], providing cells with the possibility to swiftly alter the susceptibility to induction of the mitochondrial apoptosis pathway. Furthermore, Mcl-1 has been reported to be regulated across the cell cycle and to play a particularly relevant role in (transiently) upholding apoptosis resistance during mitotic arrest prior to being degraded [39, 40]. Together with our evidence on cell cycle arrest in G2/M phases (Fig. 2D, Supplemental Fig. 8) and the function of CKIs as transcriptional inhibitors, we hypothesised that Mcl-1 downregulation might be linked to apoptosis sensitization in our scenario. We, therefore, examined Mcl-1 expression in the gliomasphere cultures following CYC065 or THZ1 treatment. CKI treatment indeed downregulated Mcl-1 protein amounts in the gliomasphere cultures in all models tested and coincided with downstream caspase-3 activation, as evidenced by accumulation of cleaved caspase-3 (Fig. 3C). Mcl-1 loss and caspase-3 activation correlated with dephosphorylation of RNA polymerase II (RNAPII) and loss of total RNAPII (Fig. 3C), which indicates suppressed transcription and previously was demonstrated to arise as a consequence of CKI treatments [18, 41]. We next tested if Mcl-1 downregulation alone might be sufficient to induce apoptosis at least in gliomaspheres expressing high amounts of this protein. Indeed, the viability of N16-0240 gliomaspheres dropped rapidly upon treatment with increasing concentrations of the highly selective Mcl-1 inhibitor, S-63845 [42] (Fig. 3D). Similarly, siRNA-based knockdown of Mcl-1 resulted in caspase-3 activation and apoptotic cell death (Fig. 3E, F, G), accompanied by sphere disintegration (Fig. 3H). Similar effects were observed in the recurrent GTCC-9 gliomaspheres (Supplemental Fig. 10A–E). We also examined the levels of Mcl-1 in a cell line that required a longer incubation time to show a response to CKIs, N15-1027 (Fig. 2H) and a cell line that was resistant to the treatment, N14-1028 (Fig. 2H). In the N15-1027 line, Mcl-1 expression was quite modest, although 120 h exposure to the CKIs did reduce these low levels of Mcl-1 further (Supplemental Fig. 11A). The N14-1208 cell line displayed particularly low basal levels of Mcl-1 and no changes were observed in Mcl-1 protein levels in this cell line upon treatment with CKIs (Supplemental Fig. 11A). In line with their modest Mcl-1 expression levels, neither cell line responded significantly to treatment with the Mcl-1 specific protein inhibitor S-63845 (Supplemental Fig. 11B). It needs to be noted, though, that such differential Mcl-1 expression across the GBM models did not indicate treatment responsiveness (Fig. 4A, B). We reasoned therefore that the decision to undergo apoptosis, therefore, must be cast by other Bcl-2 family members. Since it was previously shown that degradation of Mcl-1 during conditions of mitotic arrest paves the way for Bim-induced activation of mitochondrial apoptosis [40], we next studied the expression of this pro-apoptotic Bcl-2 family member. Bim was expressed highly heterogeneously across the gliomasphere models (Fig. 4C), and Bim amounts strongly negatively correlated with sensitivity to CYC065 and THZ1 (Fig. 4D). To further validate Bim dependency during CKI induced apoptosis in GBM, we depleted Bim expression prior to CKI-treatment (Fig. 4E). Strikingly, Bim knockdown indeed rendered GBM cells highly apoptosis-resistant upon CKI-treatments (Fig. 4F, G). To examine the potential gliomasphere-specific dependencies on other Bcl-2 family members, we analysed the expression of Bcl-2, Bcl-xL, Noxa, Bid, Puma, Bax and Bak and no correlation was found between the differential protein expression observed and subsequent cell response to CKIs (Supplemental Fig. 12).

Overall, these findings demonstrate that CKIs induce apoptotic cell death in state-of-the-art gliomasphere models primarily by Mcl-1 depletion, which then allows for Bim-dependent induction of apoptosis execution.

### CYC065 and THZ1 inhibit tumour growth in a semi-in vivo chick embryo xenograft model of primary and recurrent GBM

**Fig. 5:**
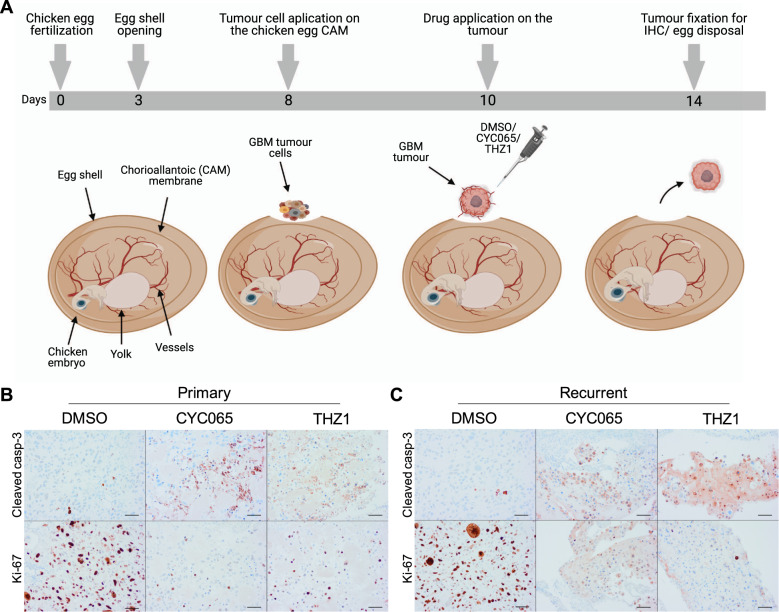
CYC065 and THZ1 are effective in a semi-in vivo chick embryo xenograft model of primary and recurrent GBM. **A** Semi-in vivo study design of the chicken xenograft model. Primary (N16-0240) and recurrent (GTCC-9) cells were implanted on the chorioallantoic membrane (CAM) of the chicken eggs (day 8 after egg fertilisation) and on day 10 treated with DMSO (control) or 3 µM CYC065 and 100 nM THZ1 for 4 days. On day 14 tumours were resected and fixed in PFA for immunohistochemical analysis. **B** Representative images of immunohistochemical staining for cleaved caspase-3 and Ki67 in the harvested tumours from the primary semi-in vivo chick embryo xenograft model established using N16-0240 cells and treated with DMSO, CYC065, or THZ1. Three tumours were collected per group. Scale bar = 50 µm. **C** Representative images of immunohistochemical staining for cleaved caspase-3 and Ki67 in the harvested tumours from the recurrent semi-in vivo chick embryo xenograft model established using GTCC-9 cells and treated with DMSO, CYC065 or THZ1. Three tumours were collected per group. Scale bar = 50 µm.

Since CYC065 and THZ1 readily induced significant levels of apoptotic cell death across the panel of gliomasphere cultures (Fig. 2A, H), we finally assessed whether these treatments could inhibit primary and recurrent GBM tumour growth also in the chick embryo xenograft models, that provide semi-in vivo data for the preclinical evaluation of antitumour agents [43]. Once tumours were established, treatments were administered and tumours were allowed to expand for a further four days (Fig. 5A). CKI treatment inhibited macroscopic tumour growth in both models upon visual examination of the excised tumours. Moreover, xenografts treated with either CYC065 or THZ1 exhibited increased cleaved caspase-3 positivity and reduced numbers of Ki-67 positive cells compared to control xenografts in both the primary (Fig. 5B) and recurrent (Fig. 5C) semi-in vivo models. Collectively, these results show that CYC065 and THZ1 when administered as single agents exert antitumour activity in semi-in vivo models of primary and recurrent GBM.

**Fig. 6:**
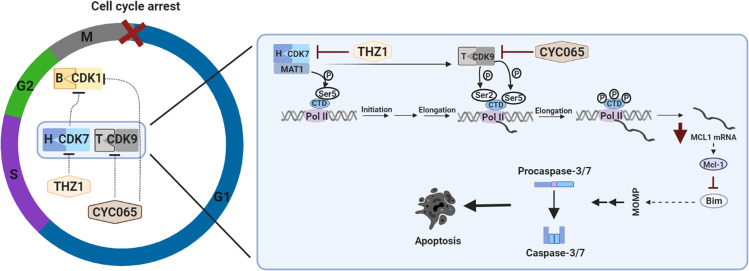
Schematic representation of cell cycle arrest and apoptotic cell death induced in CKI-sensitive cells using CYC065 and THZ1. Cell cycle progression and transcription are under the control of multiple CDKs and their associated cyclins. The CDK1/-cyclin B1 complex is necessary for the progression of the cells through the G2/M phase of the cell cycle. CDK7 is one of the main kinases required for CDK1/cyclin B complex formation and activation during the G2/M transition of the cell cycle. Failed activation of CDK1/cyclin B complex causes cell cycle arrest in the G2/M phase. Both CYC065 and THZ1 affect the CDK1/cyclin B1 complex formation, stalling cell cycle progression at the G2/M phase of the cell cycle. On the other hand, phosphorylation of the CTD of RNAP II by CDK7/cyclin H and CDK9/cyclin T complexes leads to transcription initiation and elongation, respectively. In addition, the CDK9/cyclin T complex is under the direct control of CDK7, and both are necessary for successful transcription. Inhibition of CDK9 and 7 by CYC065 and THZ1, respectively inhibit transcription through dephosphorylation of Ser2/5 on CTD of RNAPII, ultimately leading to downregulation of short-lived mRNAs such as Mcl-1. This releases pro-apoptotic Bim which triggers multiple reactions leading to mitochondrial membrane permeabilization (MOMP) and subsequent executioner caspase activation followed by apoptosis induction.

In summary, our proposed mechanism of action of the CDK-2/9-targeting CYC065 and the CDK-7 targeting THZ1 involve their dual roles in regulating cell cycle progression and transcription. In vitro and in vivo models demonstrated the ability of CYC065 and THZ1 to activate executioner caspases and trigger apoptosis in both primary and recurrent settings. Mechanistically, we have demonstrated that both CYC065 and THZ1 induced cell cycle arrest followed by caspase-dependent apoptosis. Additionally, we have shown that apoptosis occurred primarily through downregulation of the anti-apoptotic Bcl-2 protein Mcl-1, correlating with RNAPII Ser-2/5 dephosphorylation. Further analysis identified CKI-induced Mcl-1 loss as a prerequisite to establishing conditions at which the BH3-only protein Bim can efficiently induce intrinsic apoptosis (Fig. 6).

## Discussion

Identification of novel clinically relevant therapeutic strategies for primary and especially recurrent GBM remains an unmet need. Here, we provide an in-depth analysis of the effectiveness of the transcriptional CDK inhibitors, CYC065 and THZ1, targeting CDK2/9 or 7 respectively, to treat primary and recurrent GBM. We utilised in vitro and semi-in vivo models to test the effectiveness of CYC065 and THZ1 to support their possible future clinical investigation. The panel of gliomasphere cultures established in this study were representative of their parental human tumours [44–46] and an array of driver mutations were present. We demonstrate that CYC065 and THZ1 can effectively induce apoptotic death, suppress both long term survival and invasion and inhibit in vivo tumour growth of both primary and recurrent GBM, highlighting the future clinical potential of CDK inhibitors for the treatment of GBM patients.

Overexpression of CDKs 2, 9 and 7 is evident in many tumours including GBM and has been shown to inversely correlate with patient survival [31]. Indeed, specific subgroups of patients are more dependent on CDK7 (triple-negative breast cancer) [47] and/or CDK9 (MYC-driven neuroblastoma) [41]. Our analysis of GBM datasets highlighted elevated yet heterogeneous expression of CDK2, 7 and 9 in GBM patient tumours, across all subtypes (classical, mesenchymal, proneural). This is the first study to demonstrate that pharmacological inhibition of CDK2/9 or 7 can inhibit tumour growth in gliomasphere cultures regardless of their driver oncoprotein or classification as either primary or recurrent.

Our study highlights a novel approach to target GBM tumour cells specifically while sparing normal brain cells, most likely due to normal levels of RNAPII activity in non-transformed cells. Similar observations have been made in non-malignant and mammary gland breast epithelial cells treated with CYC065 [17] and THZ1-treated fibroblasts [18]. In addition, we have shown that CYC065 and THZ1 inhibit the invasive capabilities of these cultures in line with other studies [24, 48]. This is key as GBM invasion makes it extremely difficult to surgically resect, which causes high rates of tumour relapse [49]. Finally, when administrated to a semi-in vivo chick embryo xenograft model, both CYC065 and THZ1 inhibited tumour growth in primary and recurrent models. Similar results were found in rodent in vivo models of CLL, T-cell acute lymphoblastic leukaemia (T-ALL) [18], GBM [25], cholangiocarcinoma [50] using THZ1 and leukaemia model [17] and MYCN-amplified neuroblastoma [41] using CYC065.

Studies have shown that downregulation of Mcl-1 through CDK7 and 2/9 inhibition facilitates the induction of apoptotic cell death in a number of different tumour types [17, 19, 20, 41, 50]. Previous results from our lab have demonstrated that the first generation CDK inhibitor, Roscovitine, downregulated Mcl-1 in GBM, yet its potency as a single agent was limited and it was highly toxic when administered in vivo at doses required to achieve Mcl-1 downregulation [15]. On the other hand, novel transcriptional inhibitors, CYC065 and THZ1, with significantly improved potency and metabolic stability, yet retaining their ability to downregulate Mcl-1 offer great hope for their future clinical utilisation. We demonstrated here that targeting CDK2/9 and 7 using CYC065 and THZ1 lead to Mcl-1 downregulation, facilitating the apoptotic death observed in the gliomasphere cultures and in line with previous studies. Mcl-1 is of particular interest as a therapeutic target as it is one of the most frequently amplified genes across all human cancer, including GBM [51]. Mcl-1 overexpression has been shown to rescue cells from death and correlates with poorer patient prognosis in many types of haematological and some solid tumours [52, 53]. We similarly found overexpression of Mcl-1 in the TCGA GBM cohort analysed in our study. The very short half-life of Mcl-1 distinguishes it from other members of the Bcl-2 family. Due to this feature, inhibition of Mcl-1 expression and/or neutralisation of its anti-apoptotic function will rapidly make Mcl-1-dependent cells more susceptible to apoptosis and provide an opportunity to combat different types of cancers [36, 54]. However, Mcl-1 protein expression showed no correlation with the response to CYC065 and THZ1in the gliomaspheres even though the least responsive cell lines had the lowest expression of Mcl-1 among the studied cultures. Both pharmacological and genetic approaches were used to test the dependence of gliomaspheres on Mcl-1 and high Mcl-1 dependence was observed, resulting in apoptotic cell death upon CKI treatments in sensitive cells with high reliance on the Mcl-1-Bim axis.

In conclusion, in this study, we have highlighted the significant potential of two CKIs, CYC065 and THZ1 as a new treatment option for GBM. We delineate their mechanism of action, highlighting Mcl-1 as a key target of their anti-tumour activity and therefore provide further impetus for the future utilisation of these inhibitors as a novel treatment option for GBM patients in both the primary and recurrent setting.

## Supplementary information

Supplemental material Juric et al.

Supplemental Figure 1.

Supplemental Figure 2.

Supplemental Figure 3.

Supplemental Figure 4.

Supplemental Figure 5.

Supplemental Figure 6.

Supplemental Figure 7.

Supplemental Figure 8.

Supplemental Figure 9.

Supplemental Figure 10.

Supplemental Figure 11.

Supplemental Figure 12.
