# Supplementary material for: Transcriptional CDK inhibitors, CYC065 and THZ1 promote Bim-dependent apoptosis in primary and recurrent GBM through cell cycle arrest and Mcl-1 downregulation
Source: Cell Death Dis. 2021 Aug 3;12(8):763. doi: 10.1038/s41419-021-04050-7 (PMC8333061; doi:10.1038/s41419-021-04050-7)
Supplement: Supplementary file 1 — Supplemental material Juric et al. [file 41419_2021_4050_MOESM1_ESM.docx]

**Supplemental Figure Legends**

**Supplemental Figure 1. *CDK2, 9* and *7* expression does not differ between the GBM subtypes.**

*CDK2, 9* and *7* mRNA expression was studied in the GBM TCGA dataset. mRNA expression between different GBM subtypes was compared. One-way ANOVA with post-hoc Tukey’s analysis was used for statistical analysis, whereby, ns=not significant. Data from total of 528 samples was analysed.

**Supplemental Figure 2. CYC065 and THZ1 induce apoptotic cell death in commercially available GBM cell lines.**

**(A)** Cell viability in U87 and U343 glioma cell lines following increasing concentrations of CYC065 and THZ1 72 h post-treatment using WST-1 viability assay. Data are expressed as mean ± SEM.; n=3 independent experiments performed in triplicate.

**(B)** Cell morphology and death was followed upon treatment with DMSO, 3 μM CYC065 and 100 nM THZ1. Cells were pre-stained with 1μg/mL PI/Hoechst and images were taken using Eclipse TE300 inverted microscope (scale bar = 100 μm). N=3 independent experiments performed in triplicate for each condition.

**(C)** Flow cytometry was used to assess the number of AnnexinV^+^/PI^+^ cells following treatment with DMSO, 3 μM CYC065 or 100 nM THZ1 for 72 h in U87 and U343 cell lines. Data are expressed as mean ± SEM. Two-way ANOVA with post-hoc Tukey’s analysis was used for statistical analysis, whereby, ****p<0.0001; n=3 independent experiments performed.

**(D)** Flow cytometry was used to assess the percentage of cells in G0, S and G2/M phase of cell cycle. U87 and U343 cell lines were permeabilized and stained with propidium iodide after 72 h treatment with DMSO, 3 μM CYC065 or 100 nM THZ1. Data are expressed as mean ± SEM. Two-way ANOVA with post-hoc Tukey’s analysis was used for statistical analysis, whereby, *p<0.05, **p<0.01; n=3 independent experiments performed were performed

**Supplemental Figure 3. Morphology of the GBM patient-derived cultures used in the study.**

Morphological characteristics of ten patient-derived GBM cultures grown as 2-D, monolayer cultures (upper panel) or 3-D, gliomasphere cultures (lower panel). Images were taken using an Eclipse TE300 inverted microscope (scale bar = 100 μm).

**Supplemental Figure 4. CYC065 and THZ1 suppress the invasion of primary and recurrent gliomaspheres.**

(**A-D**) Primary (A, B) and recurrent (C, D) gliomaspheres were embedded in ECM in DMSO control, 3 µM CYC065 and 100 nM THZ1. Sphere invasion was followed immediately for 24 h. Images were taken using CellDiscoverer 7 (scale bar = 200 µm). Data are expressed as mean ± SEM. Two-way ANOVA with post-hoc Tukey’s analysis was used for statistical analysis, whereby, ***p<0.001, ****p<0.0001, ns=not significant; n=3 independent experiments were performed.

**Supplemental Figure 5. No correlation was observed between CKI-treatment response and CDK protein expression in gliomasphere cultures.**

Correlation analysis of protein expression (CDK2, CDK7, CDK9) in gliomasphere cultures and treatment response to 3 μM CYC065 and 100 nM THZ1 was done using Pearson correlation coefficient. N=3 independent experiments were performed.

**Supplemental Figure 6. CYC065 and THZ1 have no effect on neurite outgrowth nor viability of the mouse primary cortical neurons.**

Morphology and viability of the mouse primary cortical neurons were followed using Calcein-AM staining 96 h post treatment with DMSO, 3 μM CYC065 and 100 nM THZ1. Images were taken with an Eclipse TE300 inverted microscope (scale bar = 100 μm). N=3 independent experiments performed in triplicate for each condition.

**Supplemental Figure 7. Patient-derived gliomaspheres are highly resistant to TMZ and no synergy was observed when CYC065 or THZ1 were combined with TMZ.**

(**A**) Cell viability was measured 120 h post treatment with increasing concentrations of TMZ in patient-derived gliomaspheres using WST-1 viability assay. Data are expressed as mean ± SEM. N=3 independent experiments were performed in triplicate.

(**B, C**) Cell viability was measured 72 h after treatment with increasing concentration of CYC065 or THZ1 combined with TMZ in primary (B, upper panel) and recurrent (C, upper panel) gliomasphere cultures. Combination index values were determined by Webb’s Fractional Product method in primary (B, lower panel) and recurrent (C, lower panel) gliomasphere cultures. N=3 independent experiments were performed in triplicates. SEMs across the repeat experiments and conditions were < 10%.

**Supplemental Figure 8. CYC065 and THZ1 induce G2/M cell cycle arrest in N16-0125 and N15-0385 gliomaspheres.**

Cell cycle analysis of N16-0125 and N15-0385 gliomaspheres after treatment with DMSO, 3 μM CYC065 and 100 nM THZ1 for 48 and 72 h. Cells were fixed in EtOH, permeabilized and stained with PI. Representative histograms are shown here. N=3 independent experiments were performed in triplicates.

**Supplemental Figure 9. *Bcl-2* and *Bcl-xL* expression in GBM compared to the non-tumour tissue.**

The TCGA dataset was analysed to study mRNA expression of *Bcl-2* and *Bcl-xL* (*BCL2L1*) in non-tumour tissue and GBM patient tumour tissues. Unpaired t-test was used to determine significance, whereby **p<0.01, ns=not significant. Data from total of 538 samples was analysed.

**Supplemental Figure 10. Genetic depletion of Mcl-1 in recurrent GBM induces apoptotic cell death.**

(**A**) GTCC-9 gliomaspheres response to Mcl-1 inhibitor, S-63842 was analysed using WST-1 viability assay 72h post-treatment. Data are expressed as mean ± SEM; n=3 independent experiments performed in triplicate.

(**B**) GTCC-9 cells were transfected with scrambled control siRNA (20 nM) and Mcl-1-targeting siRNA (20 nM) for 48 h. Transfection efficiency was assessed by Western blotting and GAPDH was used as a loading control. Western blot analysis was performed in n=3 biological replicates and representative blots are shown here.

(**C**) GTCC-9 cells were transfected with scrambled control siRNA (20 nM) and Mcl-1-targeting siRNA (20 nM) for 48 h. Caspase-3 activation was followed using Western blot analysis. GAPDH was used as a loading control. Western blot analysis was performed in n=3 biological replicates and representative blots are shown here.

(**D**) Apoptotic cell death was measured using AnnexinV/PI staining in cells transfected with scrambled control siRNA (20 nM) and Mcl-1-targeting siRNA (20 nM) in GTCC-9 gliomaspheres. Data are expressed as mean ± SEM. One-way ANOVA with post-hoc Tukey’s analysis was used for statistical analysis, whereby, ****p<0.0001; n=3 independent experiments.

(**E**) Morphology changes in GTCC-9 cells transfected with scrambled control 20 nM siRNA and 20 nM Mcl-1-targeting siRNA. Images were taken with an Eclipse TE300 inverted microscope (scale bar = 200 μm). N=3 independent experiments performed in triplicate.

**Supplemental Figure 11. N14-1208 and N15-1027 gliomaspheres are resistant to Mcl-1 inhibitor, S-63845.**

(**A**) Levels of Mcl-1 in N14-1208 and N15-1027 cell lines upon treatment with DMSO, 3 μM CYC065 or 100 nM THZ1 for 120 h were followed using Western blot analysis. GAPDH is used as a loading control. Western blot analysis was performed in n=3 biological replicates and representative blots are shown here. (**B**) N14-1208 and N15-1027 gliomasphere responses to Mcl-1 inhibitor, S-63842 were analysed using WST-1 viability assay 72h post-treatment. Data are expressed as mean ± SEM; n=3 independent experiments performed in triplicate.

**Supplemental Figure 12. The expression of Bcl-2 proteins in then patient-derived gliomasphere cultures.**

Whole cell lysates from ten patient-derived gliomasphere cultures were used to determine Bcl-2, Bcl-xL, Noxa, Bid, Puma, Bax and Bak protein expression. α-Tubulin was used as a loading control. Western blot analysis is performed in n=3 biological replicates and representative blots are shown here. Correlation analysis of protein expression (Bcl-2, Bcl-xL, Noxa, Bid, Puma, Bax and Bak) in gliomasphere cultures and treatment response to 3 μM CYC065 and 100 nM THZ1 was done using Pearson correlation coefficient and R^2^ and p-values are listed in the table. N=3 independent experiments were performed.
