# Supplementary figures and images for: Transcriptional CDK inhibitors, CYC065 and THZ1 promote Bim-dependent apoptosis in primary and recurrent GBM through cell cycle arrest and Mcl-1 downregulation
Source: Cell Death Dis. 2021 Aug 3;12(8):763. doi: 10.1038/s41419-021-04050-7 (PMC8333061; doi:10.1038/s41419-021-04050-7)

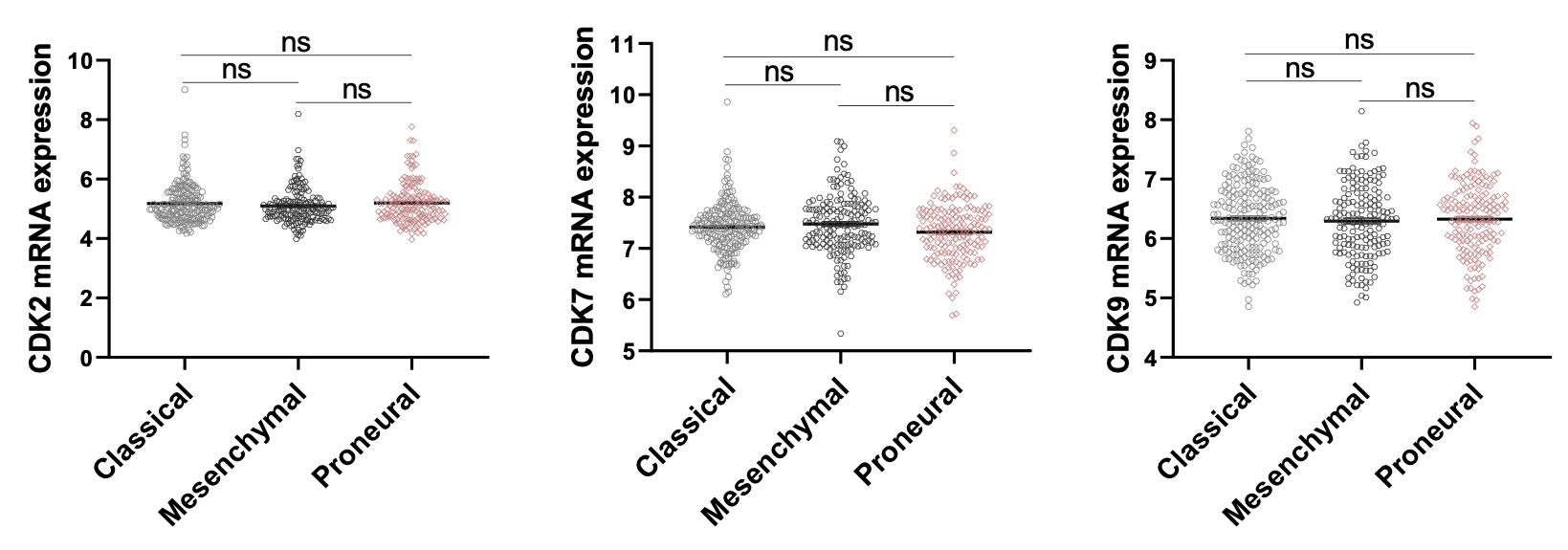

Supplement: Supplementary file 2 — Supplemental Figure 1. [file 41419_2021_4050_MOESM2_ESM.png]

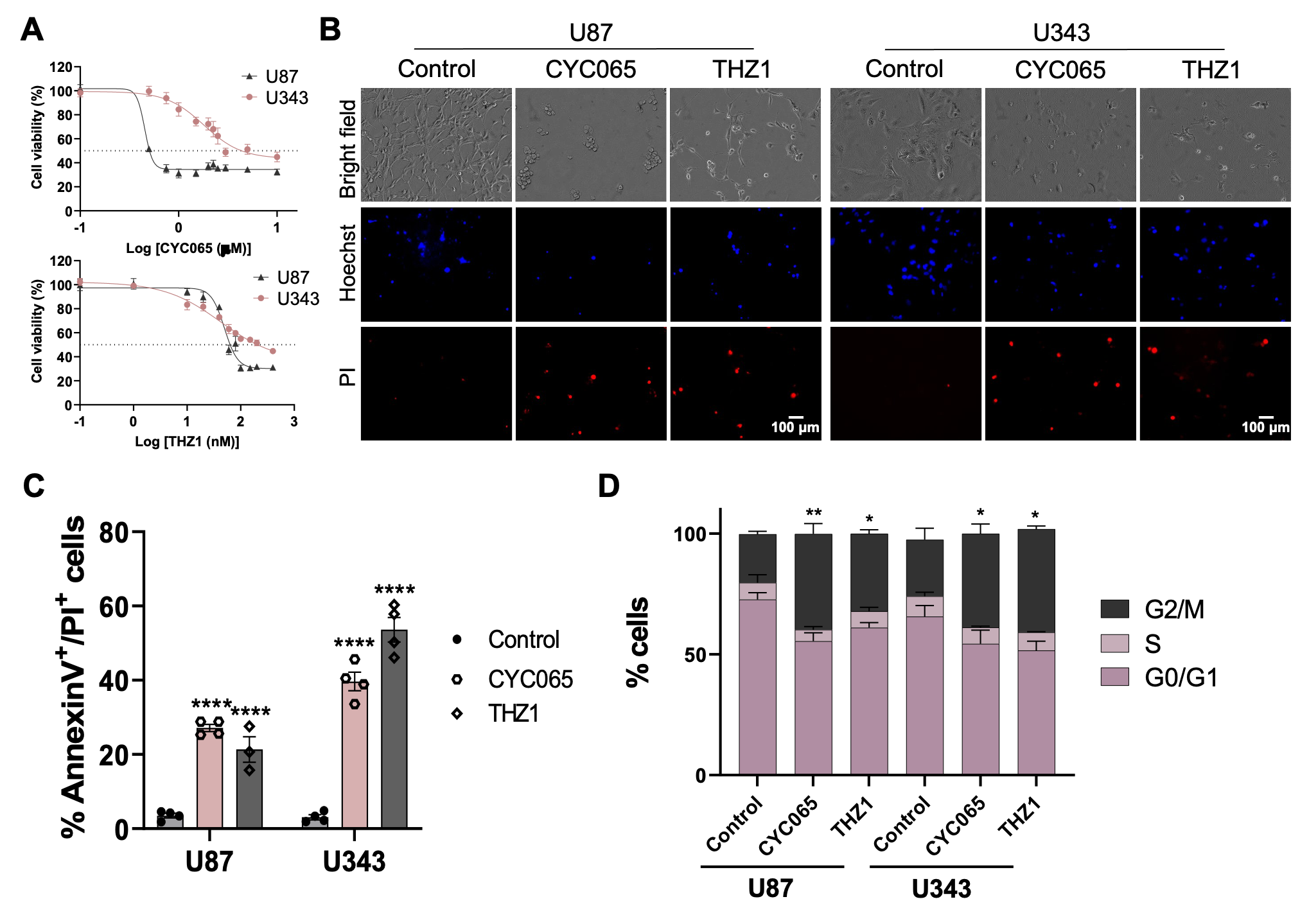

Supplement: Supplementary file 3 — Supplemental Figure 2. [file 41419_2021_4050_MOESM3_ESM.png]

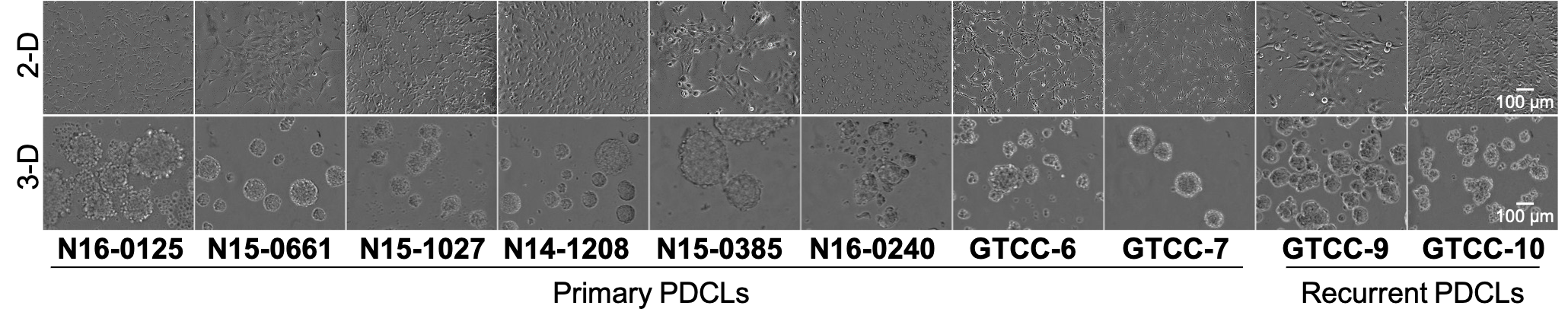

Supplement: Supplementary file 4 — Supplemental Figure 3. [file 41419_2021_4050_MOESM4_ESM.png]

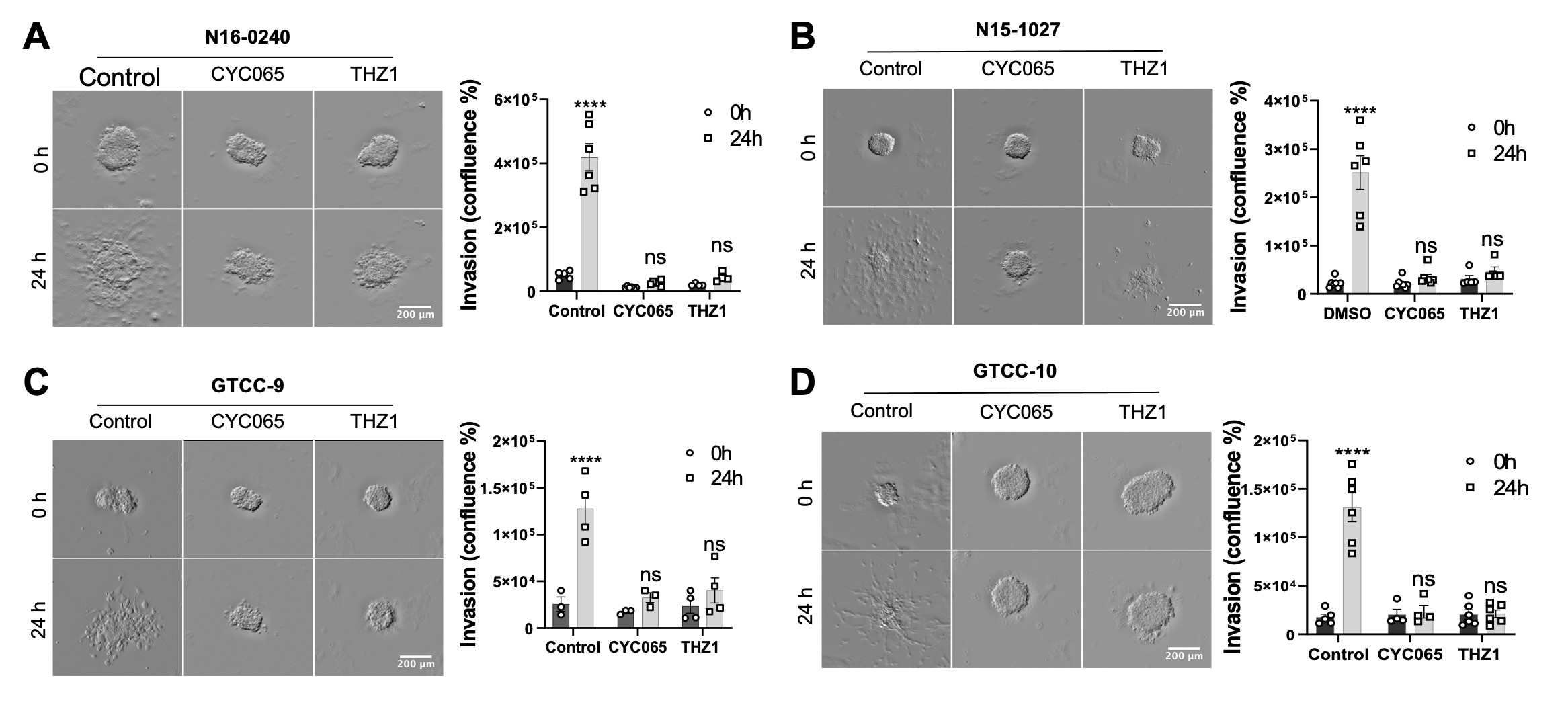

Supplement: Supplementary file 5 — Supplemental Figure 4. [file 41419_2021_4050_MOESM5_ESM.png]

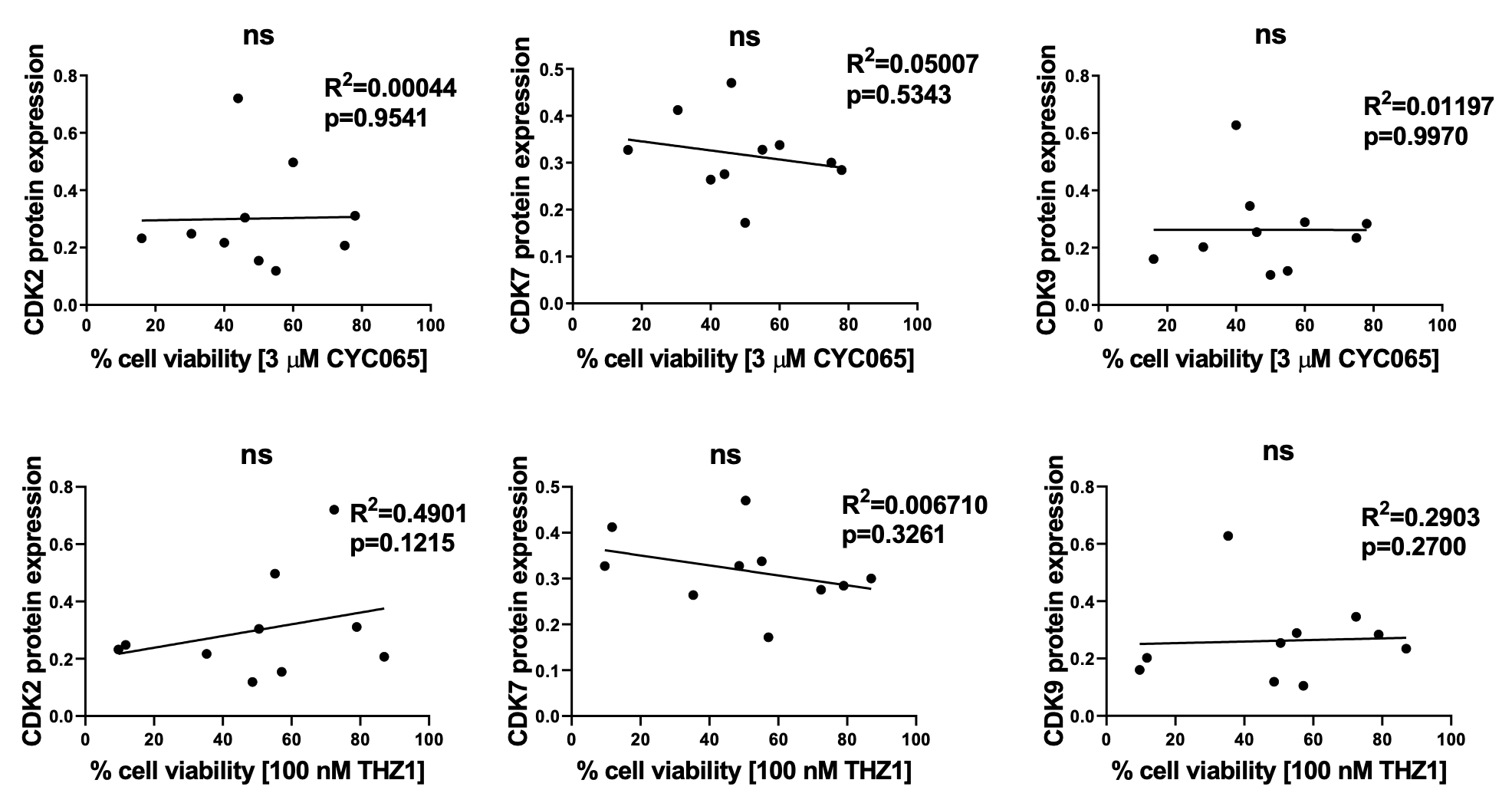

Supplement: Supplementary file 6 — Supplemental Figure 5. [file 41419_2021_4050_MOESM6_ESM.png]

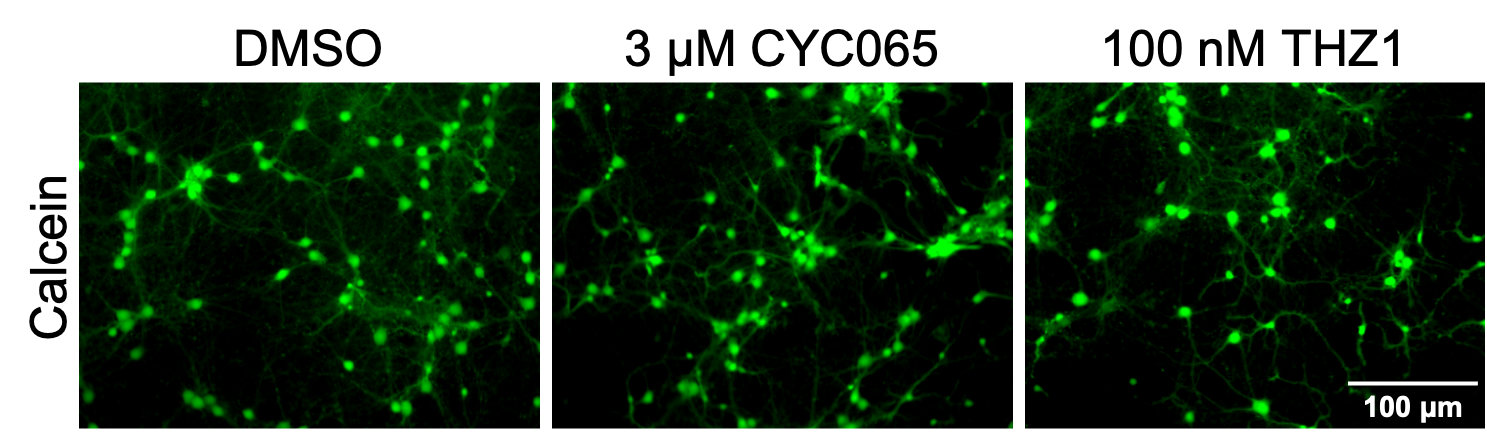

Supplement: Supplementary file 7 — Supplemental Figure 6. [file 41419_2021_4050_MOESM7_ESM.png]

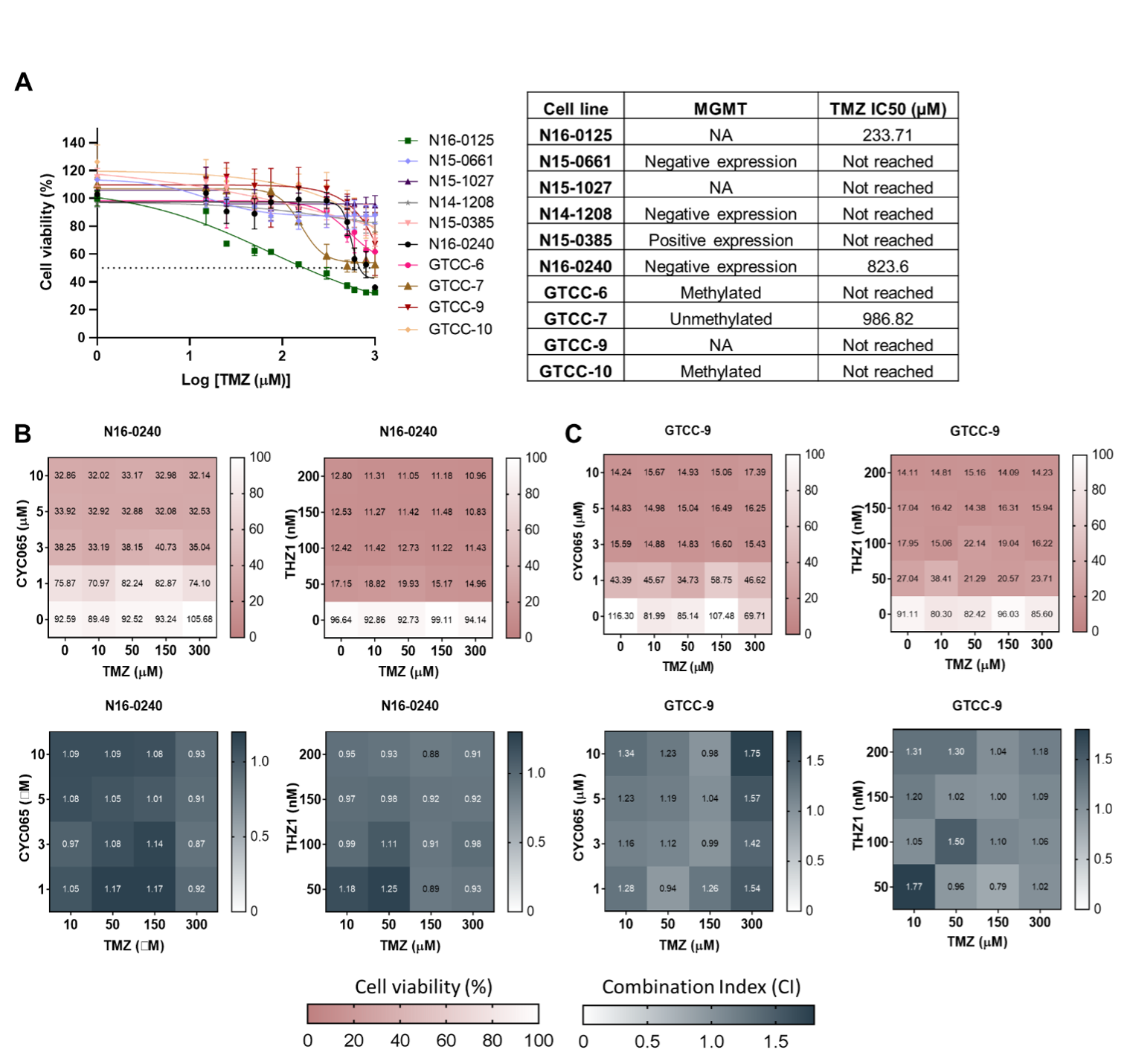

Supplement: Supplementary file 8 — Supplemental Figure 7. [file 41419_2021_4050_MOESM8_ESM.png]

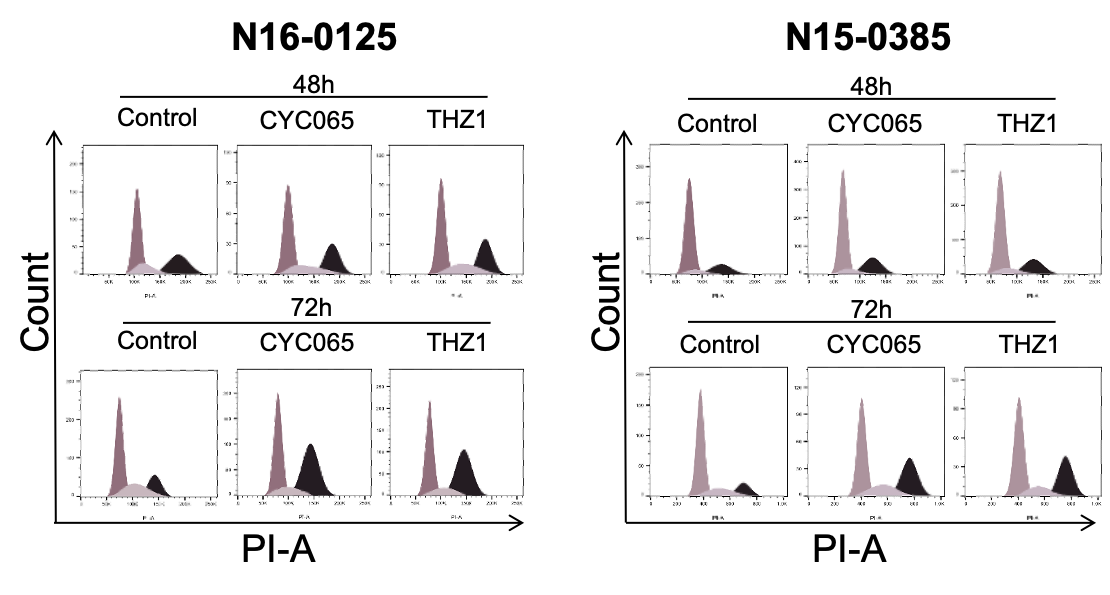

Supplement: Supplementary file 9 — Supplemental Figure 8. [file 41419_2021_4050_MOESM9_ESM.png]

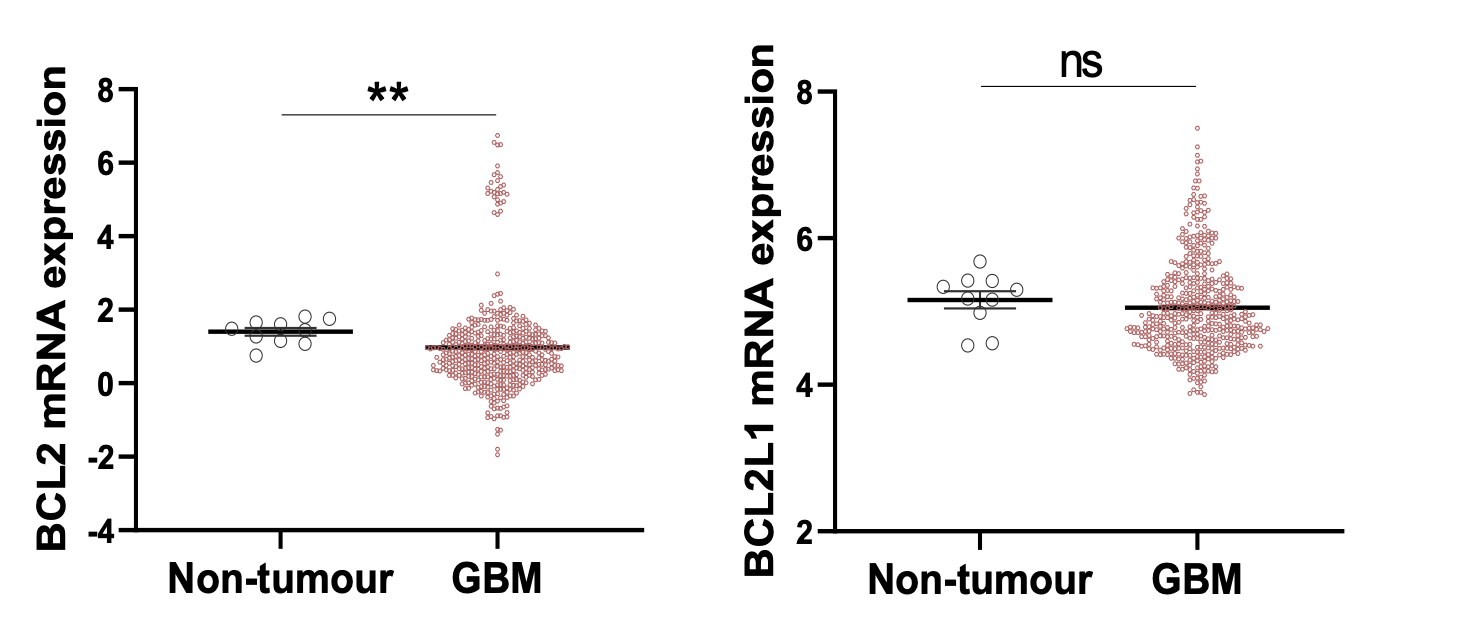

Supplement: Supplementary file 10 — Supplemental Figure 9. [file 41419_2021_4050_MOESM10_ESM.png]

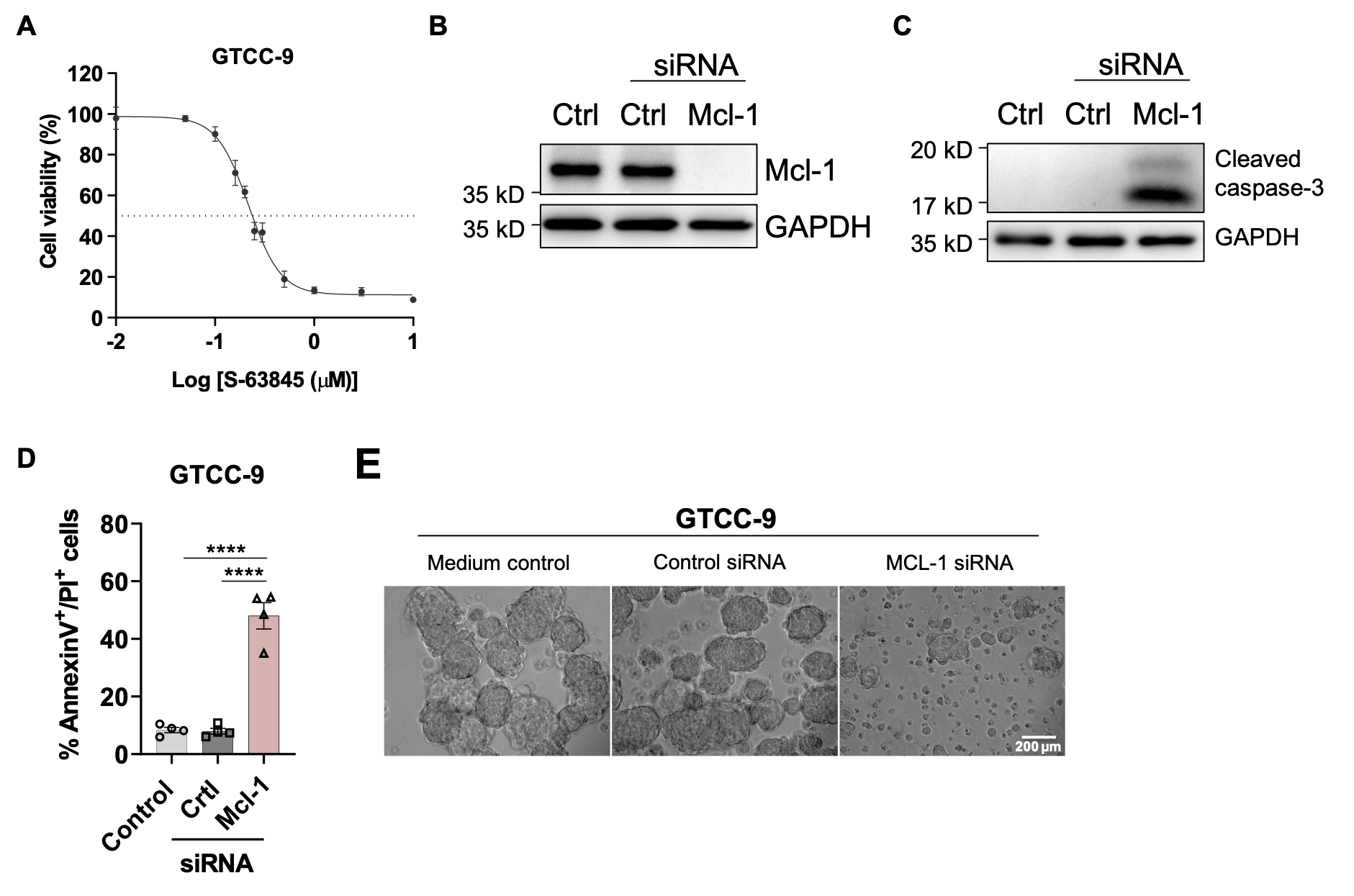

Supplement: Supplementary file 11 — Supplemental Figure 10. [file 41419_2021_4050_MOESM11_ESM.png]

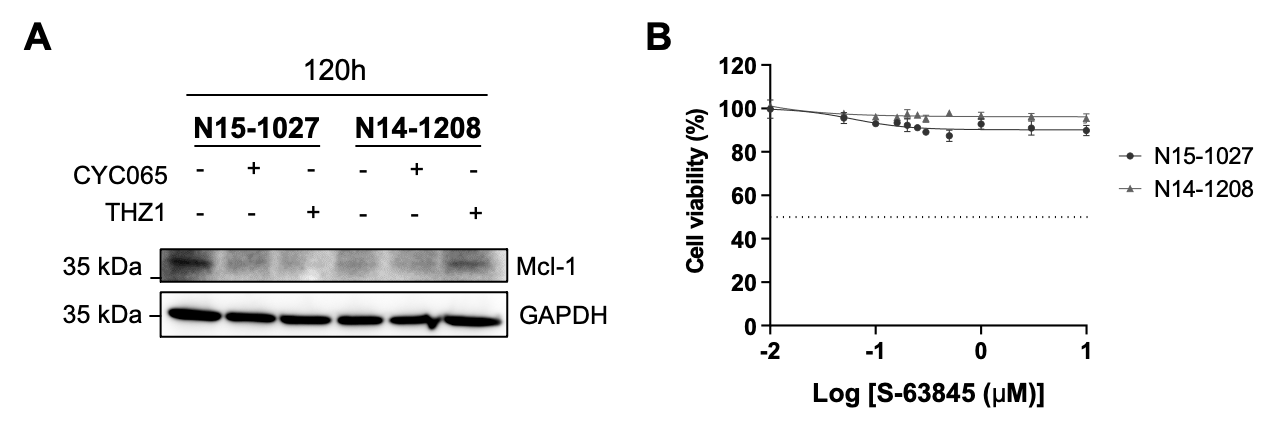

Supplement: Supplementary file 12 — Supplemental Figure 11. [file 41419_2021_4050_MOESM12_ESM.png]

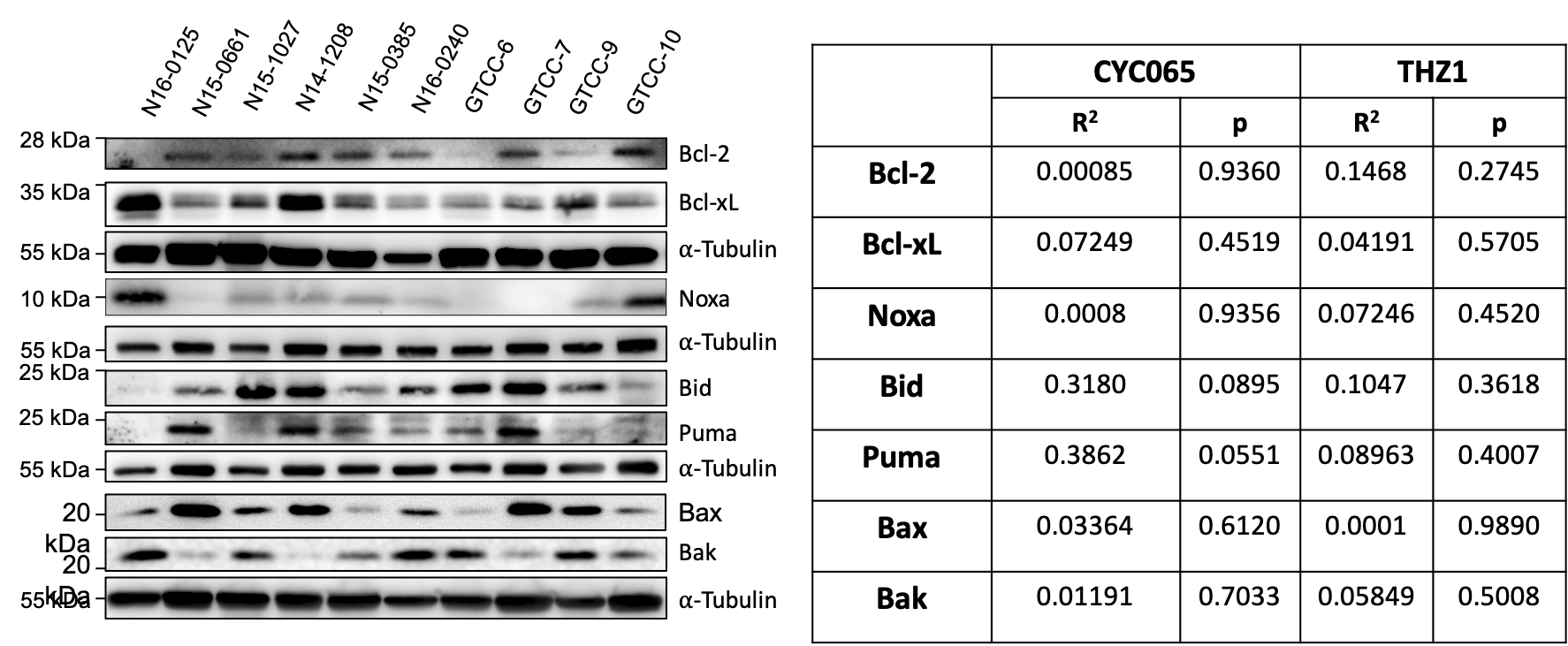

Supplement: Supplementary file 13 — Supplemental Figure 12. [file 41419_2021_4050_MOESM13_ESM.png]
